# Interneuron- and GABA_A_ receptor-specific inhibitory synaptic plasticity in cerebellar Purkinje cells

**DOI:** 10.1038/ncomms8364

**Published:** 2015-07-16

**Authors:** Qionger He, Ian Duguid, Beverley Clark, Patrizia Panzanelli, Bijal Patel, Philip Thomas, Jean-Marc Fritschy, Trevor G. Smart

**Affiliations:** 1 Department of Neuroscience, Physiology and Pharmacology, UCL, Gower Street, London WC1E 6BT, UK; 2 Wolfson Institute for Biomedical Research, UCL, Gower Street, London WC1E 6BT, UK; 3 Department of Neuroscience Rita Levi Montalcini, University of Turin, 15-10126 Turin, Italy; 4 Institute of Pharmacology, University of Zurich, Winterthurestrasse 190, Zurich 8057, Switzerland

## Abstract

Inhibitory synaptic plasticity is important for shaping both neuronal excitability and network activity. Here we investigate the input and GABA_A_ receptor subunit specificity of inhibitory synaptic plasticity by studying cerebellar interneuron–Purkinje cell (PC) synapses. Depolarizing PCs initiated a long-lasting increase in GABA-mediated synaptic currents. By stimulating individual interneurons, this plasticity was observed at somatodendritic basket cell synapses, but not at distal dendritic stellate cell synapses. Basket cell synapses predominantly express β2-subunit-containing GABA_A_ receptors; deletion of the β2-subunit ablates this plasticity, demonstrating its reliance on GABA_A_ receptor subunit composition. The increase in synaptic currents is dependent upon an increase in newly synthesized cell surface synaptic GABA_A_ receptors and is abolished by preventing CaMKII phosphorylation of GABA_A_ receptors. Our results reveal a novel GABA_A_ receptor subunit- and input-specific form of inhibitory synaptic plasticity that regulates the temporal firing pattern of the principal output cells of the cerebellum.

## Abstract (web summary)

Rebound potentiation (RP), a form of inhibitory plasticity in the cerebellum, is characterized by an increase in GABAergic synaptic currents. Here the authors demonstrate that RP is both interneuron input-specific and GABA_A_ receptor subunit-specific and serves to regulate Purkinje cell firing patterns.

## Graphical abstract

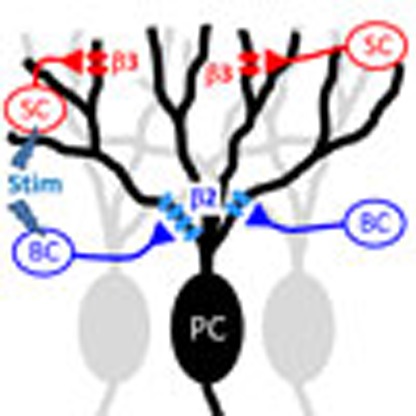

The cerebellum orchestrates coordinated movement via the spike firing patterns of its principal output neurons, the Purkinje cells (PCs)^1^,^2^,^3^. Mature PCs exhibit firing profiles that range from tonic to burst-like as well as both up- and down-membrane potential states^4^,^5^, which are thought to be influenced by the balance between inhibitory transmission and the activation of intrinsic inward currents^5^,^6^,^7^,^8^,^9^,^10^. Cerebellar synaptic inhibition is characterized by large-amplitude inhibitory postsynaptic currents (IPSCs) at basket cell (BC)–PC somatic synapses^11^,^12^, contrasting with stellate cell (SC) innervation onto distal PC dendrites, which results in generally smaller-amplitude IPSCs^13^.

PC spike firing can be dynamically regulated by inhibitory synaptic transmission, and it is likely that plasticity plays an important role in this process. Rebound potentiation (RP) is a form of cerebellar inhibitory synaptic plasticity that is characterized by increased IPSC amplitudes following PC depolarization, which can be induced either by direct current injection or by climbing fibre stimulation and synaptic AMPA receptor activation^14^. The resulting postsynaptic Ca^2+^ entry via voltage-gated Ca^2+^ channels activates Ca^2+^/calmodulin-dependent protein kinase II (CaMKII) and cyclic AMP-dependent protein kinase. These kinases can subsequently phosphorylate γ-aminobutyric acid type-A (GABA_A_) receptors (which are known kinase substrates^15^,^16^,^17^,^18^,^19^) to enhance synaptic GABA currents^20^,^21^,^22^.

The potentiation of IPSCs is likely to involve changes in the numbers of postsynaptic GABA_A_ receptors and may rely on receptor subunit composition, which can affect the outcome of phosphorylation^23^,^24^. The expression pattern of GABA_A_ receptor subunits in PCs is relatively limited compared with other neurons; only α1, β2/3 and γ2-subunits have been detected^25^,^26^. If it is assumed that different β-subunits do not co-assemble within single-receptor pentamers, this implies that synaptic GABA_A_ receptors are composed of either α1β2γ2- or α1β3γ2-subunit combinations^26^,^27^,^28^.

Here, we report that RP is an input-specific plasticity that occurs only at BC–PC inhibitory synapses and critically relies on GABA_A_ receptors containing the β2-subunit. Activation of CaMKII results in the recruitment of GABA_A_ receptors to the cell surface, whereby they reduce the spontaneous action potential firing frequency in PCs. Thus, a unique synapse- and GABA_A_ receptor isoform-specific plasticity modulates the temporal coding profile of PC action potentials. Such plasticity in the cerebellar output neurons is expected to impact on motor control.

## Results

### Induction and nature of RP

**Figure 1:**
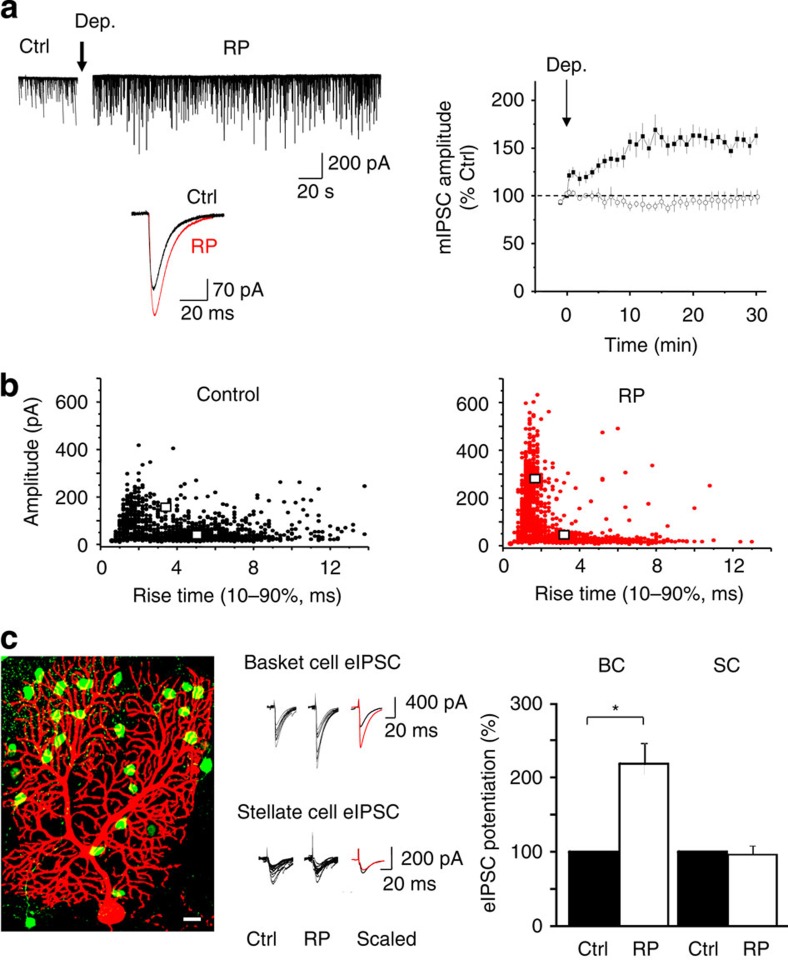
Input-specific plasticity involving basket cells. (**a**) Left, mIPSCs recorded from a single PC before (Ctrl) and after RP induction (Dep.=depolarisation step arrow). The inset depicts superimposed mIPSCs before and after RP induction. Each mIPSC is an averaged peak amplitude from 50 consecutive mIPSCs. Right, time profiles for mIPSC amplitudes in control (open symbols) and after RP induction (arrow: closed symbols). All events are normalized to mean values calculated from mIPSCs recorded over 1 min before applying the stimulus (*t*=0). All points are mean±s.e.m. (*n*=7). (**b**) Scatter plots of mIPSC amplitudes versus rise times under control conditions (left panel) and after the induction of RP (right panel) from a single representative PC. Coordinates defined by cluster analyses are shown as white squares. (**c**) Left, confocal image of a biocytin-filled PC (P18) subsequently conjugated to Alexa fluor 555 (red), and GFP-(green) expressing INs in a cerebellar slice. Scale bar, 20 μm. Middle, BC and SC eIPSCs before (Ctrl) and after RP induction. Insets: scaled average eIPSCs of 50 consecutively evoked events. Right, bar graphs of relative potentiation of eIPSCs from BC (left) and SC (right) inputs following RP induction (data are mean±s.e.m. of *n*=10, *P<0.05, paired *t*-test).

Using whole-cell voltage-clamp, miniature IPSCs (mIPSCs) were recorded in isolation from P11–20 mouse PCs in acute cerebellar slices. To induce identical forms of RP, we depolarized PCs either by stimulating climbing fibre inputs^29^ or by direct depolarization with a 0.5-Hz train of 8 × 100-ms voltage steps from −70 to 0 mV. Following stimulation, mIPSC amplitudes gradually increased over 10–15 min (Fig. 1a), and remained potentiated for over 30 min (162.8±9.4% of control (=100%); mean±s.e.m., *n*=7; Table 1)^14^,^20^,^21^. The increase in mIPSC amplitudes occurred without any change in the cell input resistance (naive cells 74.6±12.6 MΩ; post-RP 84.4±13.1 MΩ; *P*>0.05, unpaired *t*-test; *n*=5 for each). A comparable potentiation in GABA synaptic transmission was also evident by monitoring IPSPs using a K^+^ gluconate-based pipette solution in conjunction with the depolarizing protocol (Supplementary Fig. 1), indicating the plasticity was unaffected by changing the internal Cl^−^ concentration.

Miniature IPSCs can be categorized according to their rise times into fast and slow populations, with fast-rising events most likely associated with somatic BC synapses, and slower-rising IPSCs originating at distal SC synapses being subject to dendritic filtering. Cluster analysis of individual mIPSC amplitudes against rise times identified two clusters with centres at 150 pA and 3 ms, and 36 pA and 5 ms for control mIPSCs. The induction of RP had little effect on the second cluster (48 pA and 3 ms), but characteristically shifted the first to larger amplitudes and faster rise times (280 pA and 2 ms; Fig. 1b). The relative proportion and amplitude of fast rise time events (defined as <3 ms) were increased after RP, compared with the slower IPSCs (>3 ms), which remained unaffected (Supplementary Fig. 2). The fact that fast rise time, large-amplitude events were potentiated during RP suggests that synaptic events at primarily BC inputs may preferentially express this form of GABA synaptic plasticity.

### Interneuron input specificity of RP

Whether RP was input specific was investigated by selectively stimulating BC and SC inputs to PCs. Using GAD65-eGFP-expressing mice, we identified the spread of interneurons (INs) in and around the extensive dendritic arbor of the PC (see Methods). To ensure complete separation of IN subtypes, SCs were selected near the top of the PC dendritic arbor, and BCs were chosen from green fluorescent protein (GFP)-expressing cell bodies within a <40-μm radius from the PC soma (Fig. 1c). A micro-bipolar stimulating electrode was placed on individual IN soma to evoke IPSCs (eIPSCs) using a suprathreshold stimulus (up to 0.5 V, 0.5 ms). Single and also paired stimuli (separated by 100 ms) were then used to assess transmitter release from BC and SC axon terminals.

Stimulation of either class of IN reliably generated synaptic currents in PCs with latencies of 1–2.5 ms. To confirm that only single INs were activated, the stimulating electrode was moved 10 μm off soma. This markedly increased the failure rate (72±3% and 65±3%) and mean latencies for synaptic currents (14.4±0.9 and 13.3±1.3 ms) for BC and SC inputs, respectively (*n*=10; *P*<0.05, unpaired *t*-test). Mean eIPSC amplitudes for BC and SC inputs were 601±138 and 215±37 pA (*n*=10; *P*<0.05, unpaired *t*-test; Supplementary Fig. 3), respectively. As predicted, BC cell eIPSCs had faster rise times (1.09±0.09 ms) compared with SC eIPSCs (2.82±0.43 ms, *P*<0.05; unpaired *t*-test). After RP induction, BC eIPSCs were markedly potentiated to 1,361±391 pA (*n*=10, *P*<0.05, paired *t*-test, 25 min after RP induction; Fig. 1c; Supplementary Fig. 3). By contrast, SC eIPSCs remained unaffected (195±28 pA, *n*=10, *P*>0.05; paired *t*-test). Using paired stimuli with 100 ms interval, the paired-pulse ratios (PPRs) at BC–PC and SC–PC synapses were unaltered by RP induction (1.19±0.11 (BC) and 1.22±0.16 (SC), *n*=10), confirming that RP is a purely postsynaptic plasticity phenomenon.

**Figure 2:**
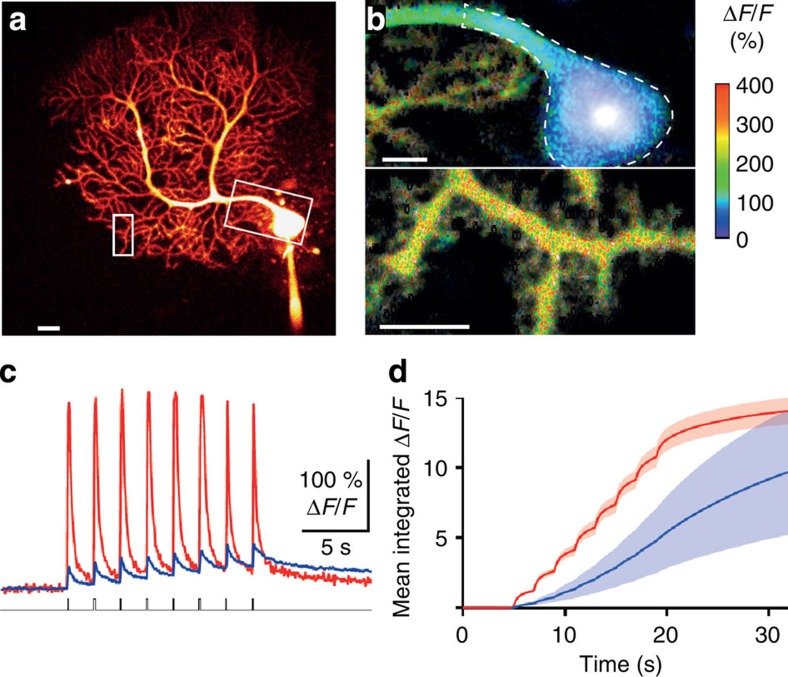
Intracellular Ca^2+^ surge in Purkinje cells during the onset of RP. (**a**) Purkinje cell filled with Alexa 594 and the Ca^2+^ indicator, Oregon Green BAPTA-1 (OGB-1), prior to RP induction. (**b**) Peak Ca^2+^ signals detected in regions of interest (ROIs) indicated by the boxes (soma and distal dendrites) shown in **a**, during a single RP induction protocol (average of 24 successive frames, three frames per voltage jump). Scale bars, 20 μm (**a**,**b**). (**c**) Relative Ca^2+^ signals (evoked by voltage jumps—upward baseline deflections, lower trace) in a dendritic spiny branchlet (red) and the soma (blue) obtained by single-line scans through the ROIs shown in **a** and **b**. (**d**) Mean-integrated Ca^2+^ signals at spiny branchlets (red) and the PC soma (blue) for three PCs (two spiny branchlets per cell are included). The shaded region denotes ±s.e.m.

The absence of RP at SC–PC synapses was not due to IPSC rundown, as all eIPSCs maintained similar amplitudes for more than 30 min during control periods of recordng. Furthermore, control mIPSCs recorded from SC–PC synapses, defined by their slow rise time (>3 ms), remained stable for over 25 min, suggesting that RP is unlikely to be occluded by any state of pre-potentiation occurring prior to RP induction (Supplementary Fig. 4). In addition, an inadequate rise in intracellular Ca^2+^ is unlikely to explain the failure to observe RP at PC–SC synapses, since the amplitudes of dendritic Ca^2+^ transients exceed those at the soma following somatic depolarization^30^. This was confirmed using two-photon Ca^2+^ imaging to detect Ca^2+^ influx in the soma and dendrites of PCs during the induction phase of RP (Fig. 2a). The RP induction protocol raised intracellular Ca^2+^ in regions of interest (ROIs) containing either proximal dendrite and soma or distal spiny branchlets (Fig. 2b), but the Ca^2+^ increase was larger in the dendrites than in the soma both in amplitude (Fig. 2c; Supplementary Fig. 5) and after integration (Fig. 2d). The possibility that the Ca^2+^ load was excessive in the dendrites and caused ‘RP occlusion' by an undefined mechanism was unlikely, given that the IPSC amplitudes at SC–PC synapses remained unaltered irrespective of whether the RP stimulus was applied. This confirmed that the lack of plasticity at SC–PC inhibitory inputs is unlikely to be a consequence of the Ca^2+^ load at dendritic locations compared with the soma where RP is readily expressed. These results indicated that RP is input specific, occurring selectively at proximal inhibitory synapses.

### β-Subunits target specific PC inhibitory synapses

**Figure 3:**
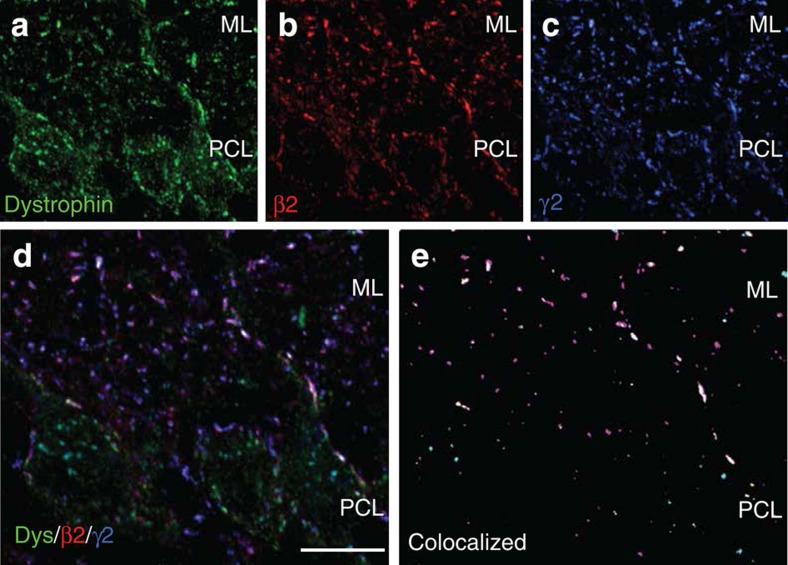
GABA_A_R β2-subunit localization in PCs. Distribution of β2-subunits in the molecular layer (ML) and Purkinje cell layer (PCL) of the cerebellum, in relation to dystrophin (a marker of GABAergic synapses on PCs) and γ2 (localized postsynaptically), as determined by immunofluorescence staining. (**a–e**) Co-localization of the β2-subunit with dystrophin immunoreactivity on PC soma dendrites (white clusters in **e**), but not on interneurons (pink clusters in **e**), revealed by triple staining with the γ2-subunit. Each staining is shown separately in **a**–**c**, and **d** depicts a projection image with superposition of the three markers. Co-localized clusters (yellow, β2/dystrophin; pink, β2/γ2; magenta, dystrophin/γ2; white, triple labelled) are shown in **e**). Scale bar, 20 μm.

**Figure 4:**
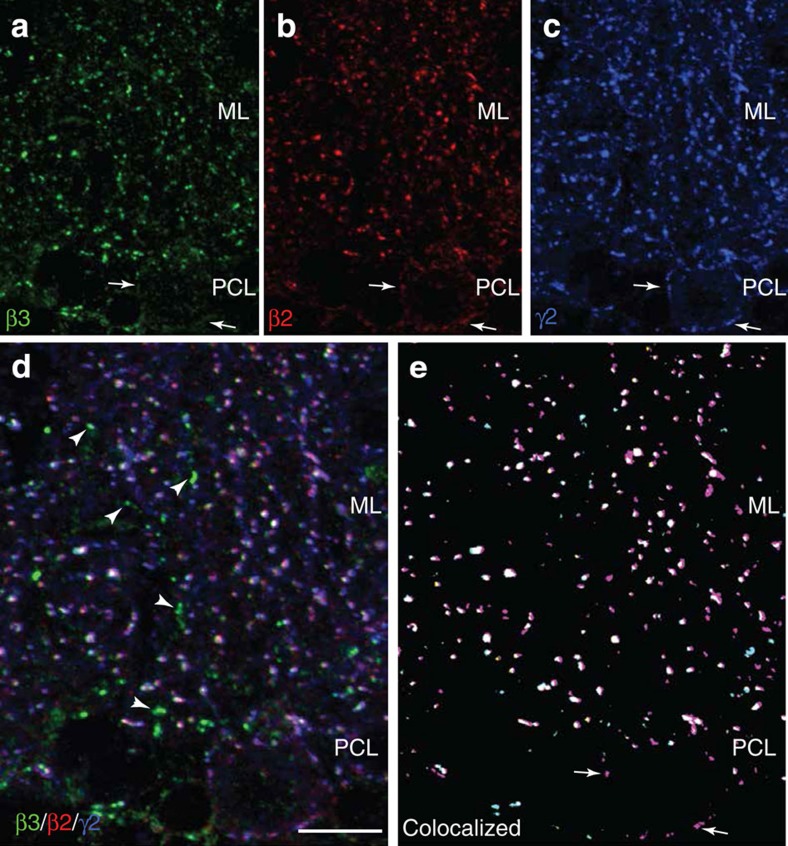
GABA_A_R β2- and β3-subunit localization in PCs. Comparative distribution of β2- and β3-subunits in the Purkinje cell and molecular layers. (**a**–**e**) Partial co-localization of the β2-, β3- and γ2-subunit immunoreactivity in presumed postsynaptic clusters located on PCs and interneurons. Individual labelling is shown in (**a–c**), and co-localized clusters in (**e**) (yellow, β2/β3; pink, β2/γ2; magenta, β3/γ2; white, triple labelled). Note the lack of β3-subunit staining around the PC soma (arrows in **a**) and in a substantial fraction of clusters in the ML; conversely, β3-subunit clusters lacking the β2-subunit (arrowheads in **d**) most likely represent GABAergic postsynaptic sites in Golgi cell dendrites (see ref. 63). Scale bar, 20 μm.

Given the limited repertoire of GABA_A_ receptor subunits that are expressed in PCs^26^,^31^, we investigated whether particular β-subunits were differentially targeted to specific inhibitory synapses and thus associated with the input-specific nature of RP. Using β-subunit-selective antibodies, the expression patterns of β2- and β3-subunits in the molecular layer was assessed in relation to the following postsynaptic markers for identifying GABA synapses: γ2-subunit, gephyrin and dystrophin (which is restricted to PCs) (Fig. 3). Using primary antibodies raised in different species, we assessed three different antibody combinations: β2/gephyrin/vesicular GABA transporter (VGAT); β2/dystrophin/γ2; and β2/β3/γ2. The first combination confirmed the postsynaptic localization of β2-subunits. The second and third combinations were used to quantitatively analyse the β-subunit distribution in GABA_A_ receptors within ROIs starting from the base of the PC soma and extending into the molecular layer (Figs 3 and 4; Tables 2 and 3). Overall, the density of postsynaptic clusters, identified by the γ2-subunit, was higher than previously reported^32^, reflecting the increased immunosensitivity that results from our new tissue preparation procedure. The β2-subunit formed numerous strongly labelled clusters containing both the γ2-subunit and gephyrin. The apposition of these clusters to VGAT-positive terminals was indicative of their postsynaptic localization (Supplementary Fig. 6).

For the β2-subunit clusters, most (77%) were co-localized with γ2, accounting for 70% of γ2-clusters. Co-staining for dystrophin (Fig. 3a–e) revealed that β2-subunits are found at PC synapses (where both markers are co-localized; Fig. 3e (white clusters)). However, β2-subunit staining was also found at IN synapses that lacked dystrophin (Fig. 3e (pink clusters)). Indeed, for γ2-subunit-containing clusters on the PC, nearly all (93%) were co-localized with the β2-subunit, whereas approximately half (47%) of postsynaptic β2-clusters were devoid of dystrophin (Table 2).

The β3-subunit also exhibited a postsynaptic clustered distribution, and was extensively co-localized (93%) with a subset of β2-subunit clusters (Table 3); interestingly, only 3% of postsynaptic β3 clusters were devoid of β2-subunits. However, of greater significance, we did not observe any β3-subunit immunoreactivity on the PC soma (Fig. 4a, arrows), despite detecting all the postsynaptic markers (Fig. 4a–e—note the lack of any marker coincident with β3 in panels d (arrowheads) and e). This may also reflect the large population of postsynaptic β2-clusters (33%) that are devoid of β3-subunits, indicating that they form a distinct somatic PC population (Table 3; Fig. 4e). Together, these results show that PCs express GABA_A_ receptors containing both β2- and β3-subunits along dendrites that correspond to SC synapses, but only express β2-subunit-containing receptors on their soma that are innervated by BC axon terminals. Thus, β2-subunit expression is relatively ubiquitous in PC cells, but β3-subunit expression is restricted to regions that do not encompass the soma. This differential distribution of GABA_A_ receptors could underlie the input-specific nature of RP.

In addition, GABAergic synapses on molecular layer INs predominantly, if not exclusively, contain β2-subunit-containing GABA_A_ receptors. The small fraction of β3 staining, devoid of co-localized β2-subunits, might be located on Golgi cell dendrites (Fig. 4).

### RP and GABA_A_ receptor isoform specificity

Given the restricted expression of β3-subunits to PC dendritic synapses, we hypothesized that the appearance of RP only at BC–PC somatic synapses is mainly supported by β2-subunits. To investigate, we used a transgenic mouse that lacks β2-subunit expression (β2^−/−^)^33^.

**Figure 5:**
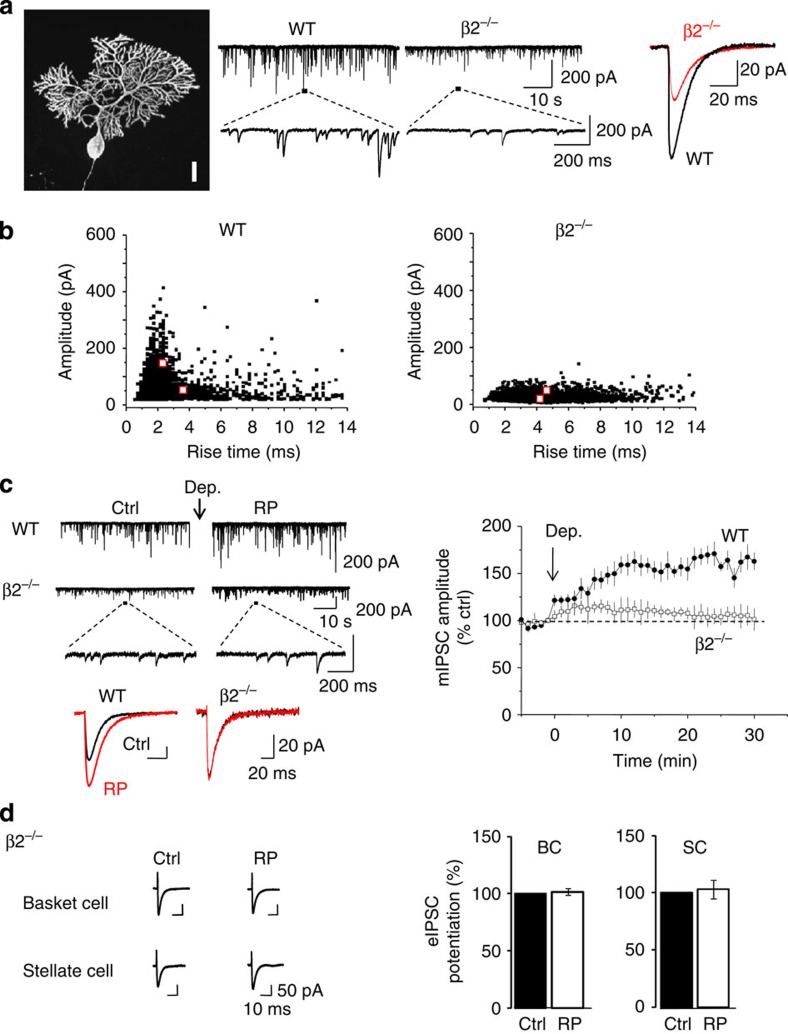
β2-subunit dependence of RP. (**a**) Left, morphology of a biocytin-streptavidin Alexa 488-filled P14 PC in a β2^−/−^ slice. Bar, 20 μm. Middle, mIPSCs recorded from WT and β2^−/−^ PCs at low and high time resolution. Right, superimposed peak-amplitude averages of 50 consecutive mIPSCs selected from WT (black) and β2^−/−^ (red) PCs. (**b**) mIPSC scatter plots of mIPSC amplitude versus rise time (10–90%) for WT (left) and β2^−/−^ (right) PCs constructed from ∼2,000 events. Cluster centres (see Methods) are shown as red squares. (**c**) mIPSCs in control and 25 min after RP induction in WT and β2^−/−^ PCs. Expanded recordings are shown below, including superimposed peak-amplitude average mIPSCs from 50 consecutive single events before (Ctrl) and after RP for WT and β2^−/−^ PCs. Right, time profiles of normalized mIPSC amplitude for WT and β2^−/−^ PCs. All points are mean±s.e.m. (*n*=5). (**d**) Left, evoked IPSCs from basket and stellate cells in a β2^−/−^ PC under control conditions and following RP induction. Right, bar graph summarizing percentage potentiation in basket and stellate cell eIPSCs after RP induction in β2^−/−^ PCs. Data are mean±s.e.m. (*n*=5).

As expected, mean mIPSC amplitudes were reduced by over 50% from 88.2±16.2 (wild-type (WT) littermates) to 41.7±9.3 pA in β2^−/−^ (*n*=5, *P*<0.05, unpaired *t*-test; Fig. 5a) and mean mIPSC frequencies reduced from 4.88±0.33 (WT) to 2.69±0.23 Hz (β2^−/−^; *n*=5, *P*<0.05, unpaired *t*-test) due to the loss of a major GABA_A_ receptor subtype. Importantly, removing the β2-subunit did not result in either a compensatory upregulation of other GABA_A_ receptor subunits (Supplementary Fig. 7; also see ref. 32). Furthermore, Sholl analysis of biocytin-filled WT and β2^−/−^ PCs indicated there were no significant changes to their morphology and neurite branching patterns (Fig. 5a; Supplementary Fig. 7).

The mIPSC amplitude distributions revealed a significant reduction in the large-amplitude IPSCs in β2^−/−^ slices, accompanied by a reduction in the fast rise time events (Fig. 5b) causing mean mIPSC rise times to increase from 1.53±0.21 (WT) to 2.59±0.06 ms (β2^−/−^; *n*=5, *P*<0.05, unpaired *t*-test). These changes are in accord with the selective silencing of the large-amplitude BC inputs in these mice. Significantly for inhibitory synaptic plasticity, RP was severely depressed in β2^−/−^ mice with only a small transient potentiation evident (115.6±4.3%, *n*=7; Fig. 5c).

To confirm the dominant role of β2-subunit-containing GABA_A_ receptors at BC–PC synapses during RP, we performed paired IN–PC recordings in β2^−/−^ slices. By selectively stimulating BCs, the resulting eIPSCs were markedly reduced to 110±40 pA in β2 ^−/−^ slices compared with WT (601±138 pA; *n*=10; *P*<0.05, unpaired *t*-test), and furthermore, RP was absent following the induction stimulus (Fig. 5d). Thus although somatic GABA_A_ receptors are seemingly lost in β2^−/−^, the residual receptors at BC–PC synapses, most likely consisting of β3-subunits, cannot support RP. By contrast, eIPSCs at SC–PC synapses were unaffected by β2-subunit deletion and remained insensitive to the expression of RP (Fig. 5d; Ctrl amplitude 177±41 pA; after RP 189±49 pA, *P*>0.05, paired *t*-test, *n*=5). This shows that the β3-subunit cannot compensate for the loss of β2 in accord with previous results from cerebellar cultures, in which GABA synaptic currents mediated by GABA_A_ receptors containing the β3-subunit were not enhanced by CaMKII but showed a small prolongation in the sIPSC decay^34^.

To determine whether any presynaptic elements contributed to RP with the β2^−/−^ PCs, the PPRs were measured, but did not vary between WT and β2^−/−^ before or after RP induction (BC and SC, Ctrl PPR 1.3±0.1; after RP 1.3±0.1; *P*>0.05, unpaired *t*-test, *n*=10).

To explore what type(s) of β-subunit remained in the β2^−/−^ slices, we took a pharmacological approach using selective reagents. If β1-subunit receptors mediated mIPSCs in β2^−/−^ slices, then a sensitivity to the β1-subunit-selective inhibitor salicylidene salicylhydrazide^35^ should be observed, but this treatment failed to affect the decay or area of mIPSCs (Supplementary Fig. 8). This contrasted with the inhibition of recombinant α1β1γ2 receptors by 1 μM salicylidene salicylhydrazide, which reduced EC_20_ GABA currents by 41±11% (*n*=3), a feature not observed with GABA currents mediated by α1β3γ2 receptors (9±6%; *n*=3). By contrast, the β2/3-selective positive allosteric modulator, etomidate^36^, prolonged the decay and increased mIPSC areas (Supplementary Fig. 8), confirming that β3-containing GABA_A_ receptors mediate mIPSCs in β2^−/−^ slices, but are unable to support inhibitory synaptic plasticity.

### CaMKII and the induction of RP

**Figure 6:**
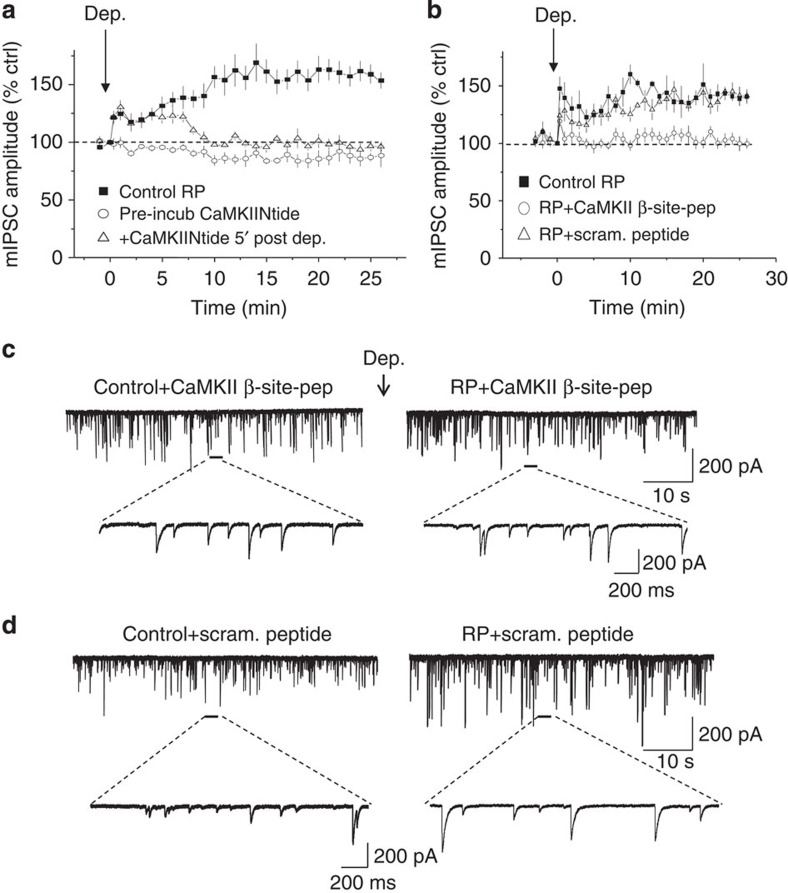
Inhibition of RP by a CaMKII-binding site peptide. (**a**) Time profiles for mIPSC amplitude before and after the induction of RP for: control RP (*n*=7); following pre-incubation in CaMKIINtide (500 nM, pre-incub, *n*=5); and CaMKIINtide applied 5 min following RP induction (post-Dep, *n*=6). All points are mean±s.e.m. (**b**) Time profiles of mIPSC amplitudes during RP under control conditions, during internal dialysis with either the CaMKII β-site peptide (*n*=7) or the scrambled peptide (*n*=4). All points are mean±s.e.m. (**c,d**) mIPSCs recorded before (left) and after (*t*=25′, right) RP induction following intracellular dialysis with CaMKII β-site peptide ((**c**) 170 μg ml^−1^) or a scrambled (Scram.) version ((**d**) 170 μg ml^−1^) as a control. Selected high-resolution mIPSCs are shown.

CaMKII is known to be important for RP induction^20^, which was confirmed using a cell-permeable form of CaMKIINtide (500 nM), a selective peptide inhibitor of Ca^2+^-independent autonomous activity of CaMKII^37^ to suppress RP (Fig. 6a). Unexpectedly, CaMKIINtide also reversed part-established RP when it was applied 5 min after PC depolarization (Fig. 6a; Supplementary Fig. 9). Notably, the conventional inhibitors KN62 (which target Ca^2+^/calmodulin binding to CaMKII) and calmidazolium (a calmodulin antagonist), have no effect on established RP^20^. These results clearly indicate that constitutive CaMKII activity is crucial for maintaining RP. We used the ability of CaMKIINtide to reverse established RP to further check whether IPSCs were pre-potentiated at SC–PC synapses prior to RP. To check this, we perfused 500 nM CaMKIINtide over 25 min during control recordings and compared the IPSC amplitude distributions generated at the start (0 min) and end (25 min) of the recording. These were unaltered, indicating the absence of a pre-potentiated state for SC–PC IPSCs (Supplementary Fig. 10).

Whether CaMKII directly interacts with GABA_A_ receptors to induce RP was addressed by creating a dominant-negative binding peptide based on CaMKII-binding site residues on GABA_A_ receptors. A highly conserved CaMKII-binding site has been identified on the major intracellular domain between M3 and M4 of β-subunits (residues 303–312). This site is located N-terminal to known kinase phosphorylation sites in β-subunits. A glutathione-linked decapeptide (CaMKII β-site peptide) matching the residues 303–312 (Gst-VNYIFFGRGP) on β-subunits showed highly specific binding to activated αCaMKII. Dialysing CaMKII β-site peptide (170 μg ml^−1^) into PCs via the patch pipette and allowing diffusion for 20 min, prevented RP (Fig. 6b,c), whereas a scrambled peptide sequence, Gst-FRNIGPFGYV (170 μg ml^−1^) did not (Fig. 6b,d).

These observations imply that a direct interaction between CaMKII and GABA_A_ receptor β-subunits is required for RP, as both β2- and β3-subunits contain consensus sites for CaMKII phosphorylation^16^,^17^,^38^,^39^.

### Increased surface GABA_A_ receptor density underlies RP

**Figure 7:**
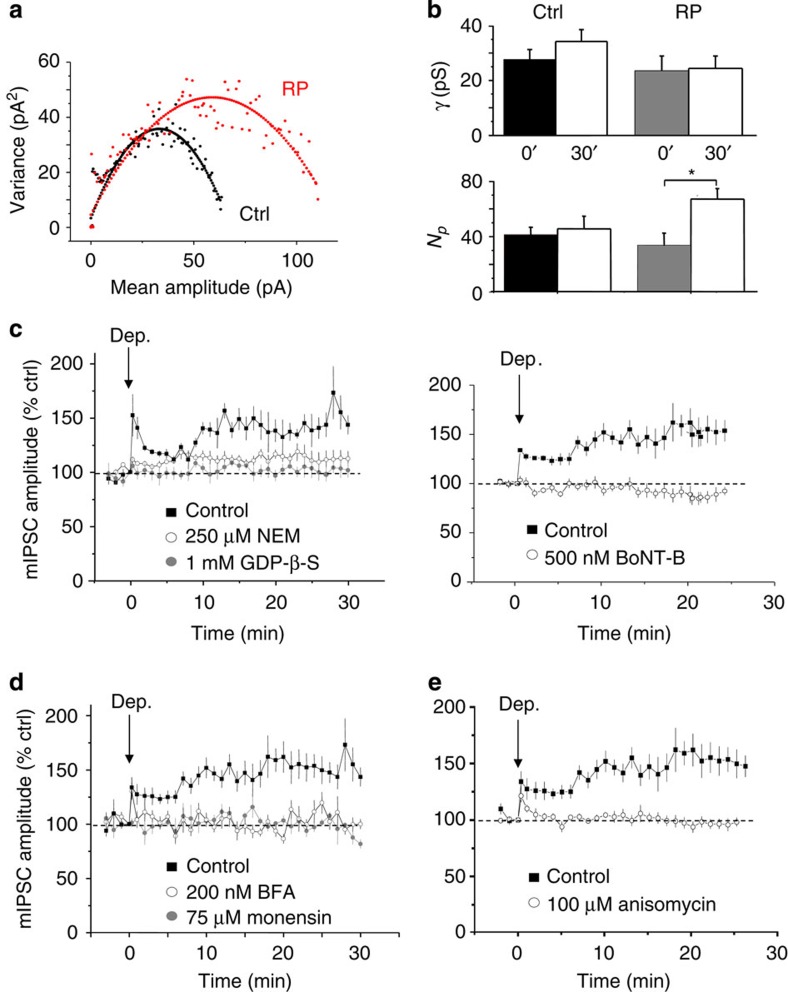
RP requires trafficking of newly synthesized receptors from the endoplasmic reticulum. (**a**) Peak-scaled non-stationary noise analysis of mIPSCs. Mean current-variance relationships under control conditions (black) and after RP (red) in a representative single PC. Consecutive single events (*n*=100) were chosen for analysis in control and 25 min after RP to construct the plots. (**b**) Estimates for the number of synaptic receptors (*N*_*p*_) and single-channel conductance (*γ*) obtained from PS-NSNA for mIPSCs under unstimulated control conditions (*n*=5) and after RP induction (*n*=7), with measurements taken at the start (*t*=0′) and end (*t*=30′) of the recording period. Data are mean±s.e.m., *P<0.05, paired *t*-test. (**c**) Time profiles of mIPSC amplitudes following the induction of RP (*n*=5), in the absence and presence of internally dialysed NEM (left, *n*=5), GDP-β-S (left, *n*=6) and BoNT-B (right, *n*=5). (**d**) Time profiles for RP induction under control conditions (*n*=5) and in the presence of monensin (*n*=5) or BFA (*n*=5). (**e**) Time profiles of RP under control conditions (*n*=5) and in the presence of anisomycin (*n*=5). All points in **c**–**e** are mean±s.e.m.

CaMKII phosphorylation of β2-subunits can enhance GABAergic transmission by increasing the number of receptors on the membrane surface, channel conductance or channel open probability. To distinguish between these possibilities, we utilized peak-scaled non-stationary noise analysis (PS-NSNA)^40^ of mIPSCs. Mean current-variance plots for both control and post-RP mIPSCs yielded parabolic relationships (Fig. 7a). Following RP, both IPSC current amplitudes and current variance increased, but the initial gradient was unaffected indicating that single-channel conductance (*γ*) for synaptic GABA_A_ receptors remained unaltered at 24.5±4.5 pS relative to control (23.7±5.3 pS; Fig. 7b, *n*=7, *P*>0.05, unpaired *t*-test). GABA channel conductance also remained constant in unstimulated PCs over a period of 30 min (Fig. 7b). By contrast, the estimated mean number of channels present in the synapse (*N*_*p*_), increased from 47.1±4.4 in control, to 69.3±5.0 after RP (measured at 30 min, *n*=5, *P*<0.05, paired *t*-test), suggesting RP involves an activity-dependent increase in the density of GABA_A_ receptors at BC synapses, which would underpin the increased IPSC amplitudes during RP.

### GABA_A_ receptor trafficking and protein synthesis in RP

To investigate whether the increase in *N*_*p*_ results from enhanced receptor trafficking into the synapse, we disrupted the fusion of intracellular receptor-containing vesicles with the cell surface membrane using *N*-ethylmaleimide^41^ (NEM, 250 μM), guanosine 5-[β-thio]diphosphate^42^ (GDP-β-S, 1 mM) or botulinum neurotoxin^43^ light-chain B (BoNT-B, 500 nM). All three inhibitors blocked the increase in mIPSC amplitude (NEM: 112.3±11.9%; GDP-β-S: 101.6±6.7%; and BoNT-B: 86.1±7.5%, *n*=5–6; Fig. 7c) and the increase in synaptic receptor numbers (*N*_*p*_) associated with RP (NEM: 42.4±13.8 and 44.8±13.3; GDP-β-S: 39.5±13.7 and 44.6±22.5; BoNT-B: 32.6±7.2 and 28.5±4.2, before and after RP induction, respectively). Basal mIPSC amplitudes were unaffected by these three inhibitors (Supplementary Fig. 11), indicating that effects on other membrane conductances were unlikely to affect the recorded IPSCs.

To establish the route by which synaptic GABA_A_ receptor numbers are increased during RP, sequentially, we disrupted intracellular receptor trafficking^44^ with internally applied monensin (75 μM) before interrupting endoplasmic reticulum/Golgi stack trafficking to the cell surface by similarly applying brefeldin-A (BFA; 200 nM) that targets GDP/guanosine triphosphate (GTP) exchange factor 2 (BIG2), which associates with GABA_A_ receptor β-subunits^45^. Each treatment prevented RP (Fig. 7d) and the increase in *N*_*p*_ (BFA: 37.4±12.1 (Ctrl), 32.6±7.6 (RP); monensin 32.8±5.3 and 33±9.9; *P*>0.05, unpaired *t*-test for each treatment versus control). Basal mIPSC amplitudes were unchanged over the 30-min recording period in unstimulated PCs in the presence of monensin or BFA (Supplementary Fig. 11).

Finally, we investigated whether RP requires *de novo* synthesis of GABA_A_ receptors. Internally applying the protein synthesis inhibitor, anisomycin^46^ (100 μM), prevented RP (Fig. 7e) and the increase in *N*_*p*_ (41.9±3.6 (Ctrl) and 42.4±9.8 (RP), *P*>0.05, paired *t*-test). Thus, RP is dependent on the synthesis of new GABA_A_ receptors and their trafficking to the cell surface membrane of PCs. However, the inhibitors, particularly anisomycin, could also affect the synthesis (even local synthesis) of trafficking factors or anchoring proteins, which, in limited supply, could also reduce numbers of cell surface GABA_A_ receptors.

### RP regulates spontaneous firing in PCs

After establishing that RP is confined to BC–PC inhibitory synapses and relies on GABA_A_ receptor β2-subunits for membrane insertion of additional receptors, we investigated the impact of RP on spontaneous firing of PCs under current clamp.

**Figure 8:**
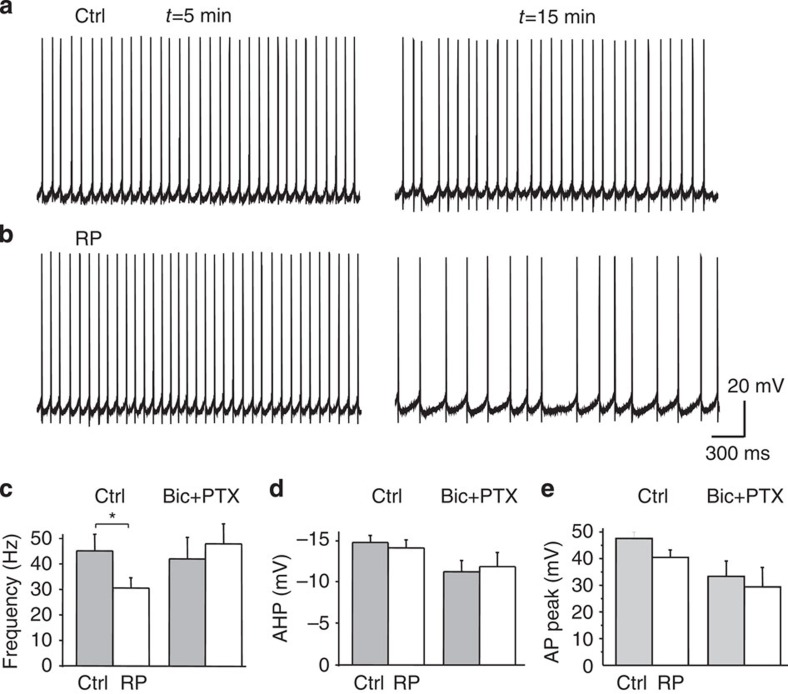
RP modulates spontaneous firing patterns in PCs. (**a**) Control current-clamp recordings of tonic spike firing in a PC, demonstrating basal spontaneous firing at 5 and 15 min after patch breakthrough. (**b**) Current-clamp recording in a PC at 5 min and then 15 min after the induction of RP. Note the increased ISIs at 15 min. (**c**–**e**) Bar graphs of spike firing frequency (**c**), spike afterhyperpolarization (AHP) amplitude (**d**), and action potential (AP) peak amplitude (**e**), before (Ctrl) and after RP induction, in the absence (*n*=5) and also in the presence (*n*=7) of bicuculline and PTX (both at 50 μM). Data are mean±s.e.m., *P<0.05. unpaired *t*-test).

Control PCs were spontaneously active in slice preparations, firing spikes continuously^4^,^5^ (Fig. 8a). PCs exhibited firing patterns and interspike interval (ISI) distributions, which were largely indistinguishable when sampled at 5 and 15 min after patch breakthrough (Fig. 8a).

**Figure 9:**
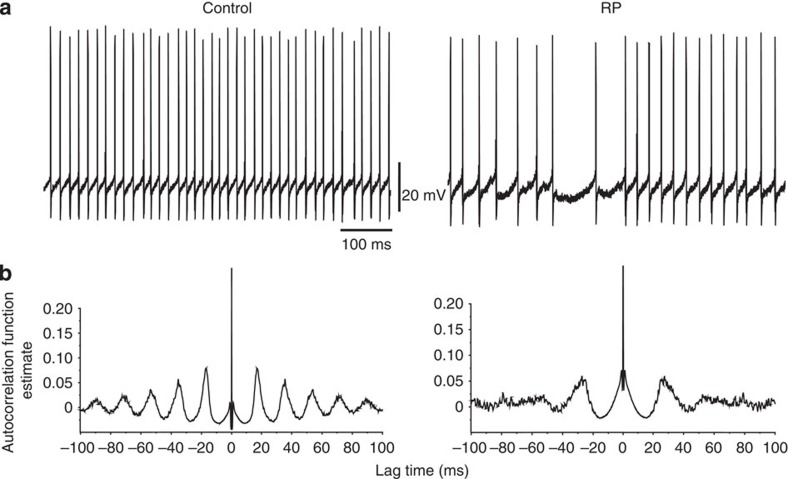
Spike periodicity during RP. (**a**) Spontaneous spike firing trains recorded from a PC in a slice under control conditions (left) and after the induction of RP (right) at a membrane potential of −62 mV. (**b**) Autocorrelograms for spike firing intervals taken from a typical PC during a control period (80 s) and for a similar period after RP induction. Note the reduced periodicity after RP.

However, sampling the ISIs over similar time periods after the induction of RP revealed a significant reduction in spike firing frequency and an increased variability in ISI (Fig. 8b). The distribution of ISIs showed a clear shift to higher values after RP induction compared with controls (Supplementary Fig. 12). This was emphasized by measuring the percentage change in the ISIs (ΔISI) from the start (5 min) to near the end of the recordings (15 min). For control PCs, ΔISI was unchanged at 0.49% (*P*>0.05, *n*=10, paired *t*-test), but increased for PCs subject to RP induction (35.5%, *P*<0.05, *n*=10). Furthermore, the spike autocorrelogram showed reduced firing periodicity (Fig. 9), while individual action potential waveforms remained unchanged before and after RP (Fig. 8d,e).

To confirm that GABA_A_ receptors underlie the reduced spike firing frequency during RP, synaptic inhibition was blocked by co-applying bicuculline (50 μM) and picrotoxin (50 μM). Baseline mean firing rate and the spike profiles were unaffected. However, antagonizing GABA_A_ receptors prevented the firing pattern changes induced by RP (Fig. 8c), leaving the mean ISI relatively unaffected by RP induction (ΔISI: 3.2%, *n*=8, *P*>0.05, paired *t*-test). Moreover, action potential waveforms were unaltered by blocking GABA_A_ receptors (Fig. 8d,e).

**Figure 10:**
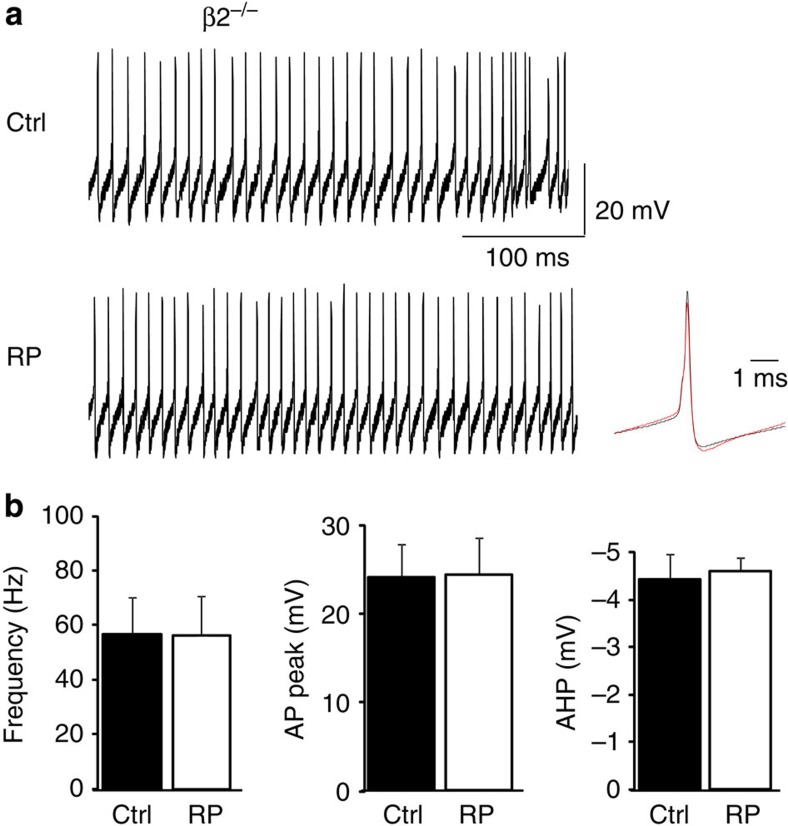
Spontaneous spike firing in β2^−/−^ mice. (**a**) High-resolution current-clamp recordings from a PC in a β2^−/−^ slice under control conditions (upper) and after RP (lower). Superimposed scaled averaged action potentials (black—control; red—RP) are shown on the right. (**b**) Bar graphs of the spike firing frequency (left), action potential (AP) peak amplitude (middle) and spike afterhyperpolarization (AHP) amplitude (right), before (Ctrl, black bars) and after RP induction (white bars) in β2^−/−^ PCs (data are mean±s.e.m., *n*=6).

We used the β2^−/−^ mice to corroborate our findings that RP shapes the spontaneous spike output of PCs, since these mice lack the necessary GABA_A_ receptor subunit composition to express RP. Notably, the firing profiles were similar throughout the recordings, indicating they were unaffected by the RP induction protocol (Fig. 10a). The mean ISIs were unchanged before and after RP (ΔISI 0.01%, *P*>0.05, paired *t*-test). Consistent with these data, action potential waveforms (Fig. 10a,b) also remained unaltered in β2^−/−^ PCs (*P*>0.05, paired *t*-test). The inability of RP to change the spontaneous spike firing patterns in the presence of GABA_A_ receptor antagonists (Fig. 8), and in β2^−/−^ PCs (Fig. 10), strongly supports a role for inhibitory synaptic plasticity as a major pathway for controlling the spike output profiles of PCs.

## Discussion

RP is the most robust example of inhibitory synaptic plasticity in the central nervous system, and yet our understanding of both its mechanism and functional role remains incomplete. Our data suggest RP is input specific, being expressed at one type of inhibitory synapse in PCs, and relies on a particular GABA_A_ receptor isoform, containing β2-subunits. Furthermore, RP has a profound effect on the spike firing profile of PCs, which is entirely dependent on GABA-mediated synaptic inhibition.

The input specificity of RP was indicated by the preferential potentiation of fast rise time large-amplitude mIPSCs, implying that RP originates at somatodendritic BC–PC synapses. This was unequivocally confirmed by selectively stimulating INs, revealing that only eIPSCs at BC–PC synapses were potentiated during RP. This precise control over the cellular location of synaptic plasticity provides an important mechanism for the dynamic regulation of PC excitability, following climbing fibre or parallel fibre activation^47^.

Previously, we had discovered that CaMKII can differentially modulate β2- and β3-subunit-containing synaptic GABA_A_ receptors enhancing mIPSC amplitudes (β2) or prolonging decay times (β3)^34^,^48^. During RP, mIPSC decay times remained unaltered, suggesting that β2 rather than β3-subunits are important for RP, which was emphasized by RP being abolished in β2^−/−^ mice.

Interestingly, the characteristic large-amplitude IPSCs were absent in β2^−/−^ PCs, suggesting β2-subunits and their location at BC synapses are vital for these events. While no upregulation or redistribution of other β-subunit isoforms was noted in β2^−/−^ mice^33^, using a pharmacological approach, we surmise the residual mIPSCs are likely to be mediated by β3- and not by β1-containing receptors.

Large-amplitude IPSCs are a unique characteristic of PCs supported by presynaptic ryanodine-sensitive Ca^2+^ stores that orchestrate multivesicular GABA release^12^. These stores are enriched in BC terminals^11^ and they govern mIPSC frequency in the absence of presynaptic firing^49^. The disappearance of large-amplitude IPSCs, coupled with a reduction in mIPSC frequency in β2^−/−^ PCs, implies that BC inputs are particularly susceptible to the deletion of this subunit. This was confirmed by a significant reduction in BC–PC eIPSC amplitudes and by the increased failure rate in β2^−/−^ PCs, neither of which were evident with SC–PC eIPSCs.

In accord with the electrophysiology, our immunohistochemistry showed a differential distribution of β2- and β3-subunits on PC soma and dendrites, with β3-subunits being expressed exclusively on dendrites^26^,^28^. We therefore conclude that BC–PC synapses contain a single population of GABA_A_ receptors that include the β2-subunit, and RP occurs specifically at this location, while β3- (as well as β2-) subunits populate SC–PC synapses and are unaffected by RP. It is unlikely that the dystrophin–glycoprotein complex plays a role in modulating RP, as it is present at both somatic and dendritic GABAergic synapses on PCs.

Although CaMKII is considered important for RP induction, by inhibiting its constitutive activity, we could reverse established RP. Furthermore, the elimination of RP, using a peptide modelled on the CaMKII-binding site on GABA_A_ receptor β-subunits, emphasized that CaMKII may directly interact with β2-subunits to initiate RP.

CaMKII-mediated potentiation of synaptic GABA_A_ receptor function could involve multiple mechanisms, but increasing channel open probability is unlikely, as GABA_A_ receptors at BC–PC synapses already have a high open probability due to receptor saturation caused by the rapid multivesicular release of GABA^50^,^51^,^52^. In addition, single-channel conductance is seemingly constant^50^; cf. (ref. 53). However, phosphorylation of β2-subunits can increase cell surface GABA_A_ receptor numbers^54^. RP increased synaptic GABA receptor numbers in PCs by approximately twofold without altering channel conductance. This mechanism was disrupted by inhibiting membrane vesicle fusion, internal receptor trafficking and protein synthesis, suggesting that RP relies on postsynaptic insertion of either *de novo* synthesized receptors via the trans-Golgi network and endoplasmic reticulum, or from a reserve receptor pool under the control of a locally *de novo* translated protein, possibly collybistin, which is known to directly bind GABA_A_ receptors^55^. The need to follow such a pathway would account for the relatively slow onset of RP. It is likely that the new GABA_A_ receptors are inserted in the extrasynaptic domain and then proceed to inhibitory synapses by lateral mobility in the plane of the membrane^56^,^57^.

Intracellular neurotransmitter receptors are inserted into the transporting vesicular membrane with their intracellular domains facing the cytoplasm, exposing consensus sites for phosphorylation to protein kinases^58^,^59^. CaMKII activation during RP could phosphorylate these sites^16^,^17^, triggering membrane insertion. Phosphorylation may also affect trafficking machinery or scaffold proteins. In particular, GABA_A_ receptor-associated protein, GABARAP, is known to undergo CaMKII-dependent conformational changes during later phases of RP^60^. Whether CaMKII-mediated phosphorylation also modulates collybistin function or the clustering of gephyrin is not yet established.

While feedforward inhibition from both BC and SC inputs can control PC responsiveness to excitatory inputs^2^,^7^, only RP at BC–PC synapses enabled inhibitory control over PC action potential initiation. This segregation of inhibitory synaptic plasticity is potentially important for the membrane domain targeting of inhibition. It is apparent that electrical coupling is most prevalent between molecular layer SCs that target PC dendrites^61^ and may enable concerted activity in the generation of dendritic calcium spikes^62^. By sparing the distal, coupled inhibitory network in favour of proximal synapses, RP could create permissive conditions for dendritic spikes and parallel fibre plasticity while increasing the efficacy of inhibition for controlling the timing of PC spike output. RP caused a decrease in spontaneous firing frequency that was dependent on GABA_A_ receptor activation, since GABA antagonists and β2^−/−^ mice abolished RP-induced changes in PC spike firing patterns. RP of inhibition at the soma is likely to reduce spiking frequency by shunting intrinsically generated depolarizing currents. This effect will be enhanced by the prolongation of ISIs imposed by the larger-amplitude IPSPs^5^,^6^,^10^.

Control of spontaneous firing rate by RP is expected to have important consequences for temporal coding by PCs, setting the dynamic range for output frequency and increasing the signal-to-noise ratio for parallel fibre-driven spikes^63^. The physiological trigger for RP in PCs is climbing fibre-triggered complex spikes^29^, which can occur synchronously in spatially organized subpopulations of PCs^64^,^65^,^66^ due to synchronization of electrically coupled modules of inferior olivary neurons. Olivo-cortical maps coincide with the projection maps of PC axons to their cerebellar nucleus targets^67^, where each nuclear cell may be inhibited by up to 400 PCs. By regulating PC output rate in functionally related microzones of the cerebellum, RP may selectively and substantially control inhibition of their downstream targets.

Indeed, during development, PCs in slices exhibit tonic firing^4^,^5^,^6^,^10^ and in some studies, progress to tonic- and burst-type firing patterns (trimodal) around P10–11, coinciding with the expansion of the dendritic arbor^5^,^68^. Thus, RP may be instrumental in the development of more sophisticated spike firing patterns, facilitating PC function for advanced motor execution with GABA_A_ receptors, acting as the major conduit by which RP affects firing.

The main purpose of RP could be to modulate the dynamic range of PC spike firing patterns, regulating the level of inhibition of the deep cerebellar nuclei. Given the importance of the mossy fibre–PC–deep cerebellar nuclei circuit for detecting and correcting motor behaviour^3^, RP is likely to be an important factor shaping the output of the cerebellum, contributing to the correction of motor errors, and thus playing a role in motor memory formation and consolidation. Our observations represent the first example of inhibitory synaptic plasticity in the central nervous system that exhibits not just precise interneuronal input selectivity, but a reliance on a specific GABA_A_ receptor subunit composition.

## Methods

### Slice preparation

Parasagittal cerebellar slices (250 μm) were cut from P11–20 male and female C57BL/6J and 129/SvEv mice in artificial cerebrospinal fluid (aCSF, 2–4 °C) using a Leica Vibratome VT1000s slicer. The aCSF contained (mM): 85 NaCl, 2.5 KCl, 1 CaCl_2_, 2 MgCl_2,_ 1.25 NaH_2_PO_4,_, 25 NaHCO_3_, 75 sucrose, 25 glucose, bubbled with 95% O_2_/5% CO_2_. Slices were incubated at 35 °C for 45 min, when the bathing solution was slowly exchanged over 20 min to aCSF containing (mM): 125 NaCl, 2.5 KCl, 2 CaCl_2_, 1 MgCl_2_, 1.25 NaH_2_PO_4_, 25 NaHCO_3_ and 25 (or 10) glucose, 95% O_2_/5% CO_2_. Slices were kept at room temperature (22–24 °C), before being transferred to a Nikon Eclipse E600FN upright microscope for electrophysiology.

### Electrophysiology

Inhibitory synaptic currents were recorded with a Multiclamp 700B (Molecular Devices) amplifier. mIPSCs were filtered at 10 kHz (8-pole Bessel) before being digitized (Digidata 1322A). PCs were voltage clamped at −70 mV using a patch pipette (2–3 MΩ) filled with a solution containing (mM): 150 CsCl_2_, 1.5 MgCl_2_, 0.5 mM EGTA, 10 HEPES, 2 Na-ATP, 0.4 Na-GTP, 5N-(2,6-dimethylphenylcarbamoylmethyl) triethylammonium bromide (QX-314, to block Na^+^ action potentials), pH 7.3 and 295 mOsm. Series resistance (*R*_S_) was ∼8–12 MΩ and compensated by up to ∼75%. Any PC showing more than a 10% change in *R*_S_ was discarded. For current-clamp recording, the pipette solution contained (mM): 120 K-gluconate, 9 KCl, 10 KOH, 3.48 MgCl_2_, 10 HEPES, 4 NaCl, 4 Na_2_-ATP, 0.4 Na-GTP and 17.5 sucrose, pH 7.25. The aCSF lacked receptor/channel blockers to enable synaptic inputs and regenerative spiking activity. For anatomical reconstructions of PCs, 5 mg ml^−1^ biocytin was included in the pipette solution. mIPSCs were usually recorded at room temperature (22–24 °C) in an aCSF supplemented with 50 μM D-(–)2-amino-5-phosphonopentanoic acid (D-AP5) and 10 μM 2,3-dihydroxy-6-nitro-7-sulfamoyl-benzo[f]quinoxaline-2,3-dione, to inhibit NMDA and AMPA receptors, respectively. Tetrodotoxin (0.5 μM) was used to block Na^+^-dependent action potentials. Recording of spontaneous spike firing in PCs was performed at 33–35 °C in current-clamp mode, without d.c. current injection. RP was induced by 8 × 100-ms voltage steps, from −70 to 0 mV delivered at 0.5 Hz (8 stimuli over 16 s).

Gst-CaMKII β-site peptide and the scrambled version were dissolved in the patch pipette solution (170 μg ml^−1^) and internally dialysed into PCs, whereas CaMKIINtide (Calbiochem) was bath applied. BoNT-B (Listlabs), GDP-β-S (Sigma), NEM (Sigma), monensin sodium salt (Sigma), brefeldin-A (Sigma) and anisomycin (Tocris) were individually applied by dialysis from the patch pipette and allowed to equilibrate based on their estimated diffusion coefficients (for example, 12 min for NEM and GDP-β-S, 20 min for BoNT-B).

### IN–PC single-input stimulation

IPSCs were evoked by direct stimulation of single visually identified INs. Stimulation electrodes were fabricated by inserting a bipolar electrode into the two barrels of a theta glass patch electrode (TGC150-10, Harvard) filled with aCSF. The stimulating electrode was used in ‘on-cell' loose patch mode to directly stimulate IN soma. Minimal intensity stimuli (0.5 ms) were used and the voltage slowly increased up to 0.5 V until an IPSC was evoked. Thereafter, the stimulation intensity was kept constant throughout the recording. GFP-expressing cerebellar INs from GAD65-eGFP mice were visualized using epifluorescence and differential interference contrast (DIC) optics. BC and SC were distinguished by their relative location in the molecular layer compared with PCs. Only the outermost SCs were stimulated to avoid contamination with ‘ambigious' BCs. INs within a radius of 40 μm from the soma were classified as BCs. This method allowed selective stimulation of specific presynaptic inputs. On average ∼60% of IN–PC pairs showed synaptic connectivity.

### Ca^2+^ imaging

PCs in parasagittal cerebellar slices from C57BL/6 mice (P29) were filled for at least 15 min via a patch electrode with Alexa 594 (15 μM) and OGB-1 (200 μM) in CsCl-based internal solution (see above). Cell morphology and RP protocol-induced Ca^2+^ transients were imaged using a custom-built 2-photon microscope (Prairie Technologies) and a Ti-Sapphire laser (Spectra-Physics) tuned to 840 nm. Calcium imaging was carried out at ROIs both in line-scan (100 Hz) and frame-scan (0.5 Hz) modes using Scan Image.

### Transgenic mice

GABA_A_R β2^−/−^ mice were generated by the deletion of exons 6 and 7 of the β2-subunit gene by homologous recombination^33^. Generation >F10 mice were used to produce β2^−/−^ homozygotes. The GAD65-eGFP mouse line was a gift from Michael Hausser (UCL)^69^.

### Analysis of synaptic currents

IPSCs were analysed offline using MiniAnalysis (Synaptosoft) version 6.0.3 by importing Axon binary files from Clampex 9.2. mIPSC amplitudes were normalized to the mean amplitude measured over a 1-min epoch before depolarization to induce RP. The extent of RP was calculated as the percentage change in mean mIPSC amplitudes after the depolarizing stimulus until the end of the whole-cell recording. The mIPSC rise times were measured between 10 and 90% of the peak mIPSC current amplitude. The decay time was fitted by a single exponential applied to the decaying phase of the mIPSCs. Mean values were determined from 50 consecutive single events. The spike firing rates were analysed offline using WinEDR V3.1.9 program (Strathclyde Electrophysiology Software, courtesy of Dr J. Dempster).

The mIPSC time stability profiles, amplitude histograms and Gaussian distribution fits were all analysed using Origin ver 6.0. All values are mean±s.e.m. Statistical significance was assessed using paired or unpaired Student's *t*-tests for comparing two groups. For current-clamp recordings, the parameters for spike firing frequency and ISI were averaged from tonic spike firing in PCs.

For the cluster analysis of sIPSC amplitudes and rise times, we used both an unsupervised hierarchical cluster analysis as well as a *k*-means cluster analysis based on Ward's method. We used *z*-score normalization, and the gaps between data points were calculated using Euclidian squared distances (SPSS ver 14)^34^.

Autocorrelation analyses were applied to continuous periods (∼80 s) of spiking to examine for cycles of activity in PCs. The number of data shifts used was 1,000, giving a lag time of 20 ms. The autocorrelation function estimate was then plotted against the lag time periods (using ClampFit 10.2) for control spiking periods and after the induction of RP.

### Peak-scaled non-stationary noise analysis (PS-NSNA)

One hundred single events were chosen randomly before and 25 mins after RP induction (or 0′ and 25′ during unstimulated recording). PS-NSNA was performed on a fixed length of mIPSC decay, starting from the peak current to the end of the mIPSC decay. The average current was scaled to the peak of the individual mIPSC amplitudes, and the amplitude was divided into equal-size bins. The fluctuations due to channel opening and closing during the decay were found by subtracting the mean current variance from the mean mIPSC current decay. The amplitude variance within each of the scaled average bins was then calculated and plotted against the average bin current^50^. The variance against mean amplitude relationship was fitted by:


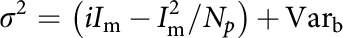


where *σ*^*2*^ is the current variance, *i* is the unitary current, *I*_m_ is mean current and *N*_*p*_ is the average number of receptor channels open in response to a single vesicle release in the synapse. Var_b_ is the baseline variance. This plot estimated single-channel current (pA) from the initial gradient of the parabola, which was converted to unitary conductance by:


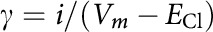


where *γ* is the unitary conductance, *V*_*m*_ is the holding potential of the cell, *E*_Cl_ is the Cl^−^ reversal potential and *i* represents the single-channel current.

### PC anatomical profiles

Slices containing biocytin-filled PCs were transferred into 4% w/v paraformaldehyde (PFA) solution and kept at 4 °C. PFA was removed by washing (3 × ) in PBS. The slices were then permeabilized with 0.4% w/v Triton-X for 1 h. After removing Triton-X by washing with PBS (3 × ), the slices were incubated for 2 h in 0.5–1 mg ml^−1^ Streptavidin-AlexaFluor 488 (or Streptavidin-AlexaFluor 533 for the dual visualization of PCs and INs) before washing with PBS (3 × ) and mounting on microscope slides with Vectashield. A Zeiss laser-scanning confocal microscope (LSM 510 Meta) was used to view the images of filled PCs. To reconstruct the PC dendritic arbor, optical sections (Z stacks) of 0.45 μm thickness (pinhole size was set to 1 AU) were transversely taken through the cell. These images were subsequently combined to project the entire arbor of single PCs. Sholl analysis was performed using 3-μm concentric circles surrounding the cell soma.

### Immunohistochemical staining

Tissue preparation was performed using a protocol that maximizes the detection sensitivity of antibodies against postsynaptic proteins^70^. Briefly, adult male C57BL/6J mice were deeply anesthetized with sodium pentobarbital (Nembutal; 50 mg kg^−1^ intraperitoneally) and perfused intracardially with 15–20 ml ice-cold, oxygenated aCSF. They were decapitated immediately thereafter and the cerebellum rapidly isolated, plunged into ice-cold, freshly prepared fixative (4% PFA in 0.1 M sodium phosphate buffer, pH 7.4) and post-fixed for 90 min, rinsed with PBS, cryoprotected overnight in 30% w/v sucrose in PBS, frozen with powdered dry ice and stored at –80 °C. Parasagittal sections were cut with a sliding microtome and processed free-floating for triple immunofluorescence staining using the following antibodies: rabbit anti-β2-subunit (Millipore, AB5561; 1:3,000), mouse anti-β3-subunit (NeuroMab, 75149; 1:1,000), guinea pig anti-γ2-subunit (University of Zurich, Switzerland; 1:10,000), mouse anti-gephyrin (Synaptic Systems, mAb7a 147-011; 1:1,000), mouse anti-dystrophin (rod domain; Leica Biosystems, NCL-Dys1; 1:50) and rabbit anti-VGAT (Synaptic Systems; 131003; 1:300). The following secondary antibodies were used (Jackson Immunoresearch); donkey anti-mouse coupled to AlexaFluor 488 (715-545-150; 1:1,000), donkey anti-rabbit coupled to Cy3 (711-165-152; 1:1,000) and donkey anti-guinea pig coupled to Dylight 649 (706-605-148: 1:300). Sections were mounted and protected with coverslips using a fluorescence mounting medium (Dako).

### Confocal microscopy and image analysis

Images were acquired with a confocal microscope (LSM510 and LSM700, Zeiss) using a planfluor oil immersion × 40 objective (numerical aperture 1.4; pixel size 90 nm), using sequential scanning of each fluorochrome to avoid bleed-through. Images were processed for visualization using either ImageJ or Imaris software (Bitplane, Switzerland). Quantification of postsynaptic clusters was performed using a custom-made macro in ImageJ, applying density threshold segmentation algorithms to isolate clusters of interest and their co-localization patterns in confocal images. Sholl analysis was performed using ImageJ.

## Additional information

**How to cite this article**: He, Q. *et al*. Interneuron- and GABA_A_ receptor-specific inhibitory synaptic plasticity in cerebellar Purkinje cells. *Nat. Commun.* 6:7364 doi: 10.1038/ncomms8364 (2015).

## Supplementary Material

Supplementary Information Supplementary Figures 1-12 and Supplementary Tables 1-3

## Figures and Tables

**Table 1:** Properties of IPSCs before and after rebound potentiation.

**IPSC property**	**Control**		**RP**	
	**0′**	**30′**	**Pre**	**Post 25′**
Amplitude (pA)	88.2±15.9	84.9±10.5	75.5±6.2	120.9±11.9*
Frequency (Hz)	4.9±0.3	5.0±0.5	5.7±1.5	5.3±1.3
Rise time (ms)	2.0±0.2	2.8±0.4	1.2±0.2	1.5±0.1
Decay time (*τ*_W_, ms)	10.3±0.8	16.1±1.8*	10.9±0.5	12.3±0.8

IPSC, inhibitory postsynaptic current; RP, rebound potentiation.

Properties of IPSCs recorded from PCs under control conditions and also before (pre) and after (post 25′) the induction of RP. Data are mean±s.e.m. (*n*=7).

^*^Indicates *P*<0.05 (unpaired *t*-test) for RP values from the equivalent timed measurements in control.

**Table 2:** Quantification of dystrophin, **β**2- and **γ**2-subunit clusters.

**Marker**	**γ2**	**β2**	**Dys**	**Dys-β2**	**β2-γ2**	**Dys-γ2**	**Triple**	**β2/γ2 No Dys**	**Dys/γ2 No β2**
Mean/1,000 μm^2^	142±13	133±12	69±7	54±8	98±11	54±6	51±7	47	3

**Marker**	**β2–γ2/γ2**	**Dys–γ2/γ2**	**Triple/β2–γ2**	**Triple/dys–γ2**	**β2–γ2 or Dys–γ2/γ2**
% Denominator	69	42	56	93	71

Top row shows the density of clusters formed in the molecular layer by each marker, whether alone or co-localized with one or two other markers, as indicated (mean±s.d., *n*=5 mice). Bottom row shows the fraction of clusters containing the indicated co-localization of markers relative to the marker in the denominator. Clusters containing the γ2-subunit are considered to be postsynaptic.

**Table 3:** Quantification of **β**2-, **β**3- and **γ**2-subunit clusters.

**Marker**	**γ2**	**β2**	**β3**	**β2/β3**	**β2/γ2**	**β3/γ2**	**Triple**	**β2γ2 No β3**	**β3γ2 No β2**
Mean/1,000 μm^2^	137±28	123±15	96±26	64±14	90±16	61±16	58±14	33	3

**Marker**	**β2–γ2/γ2**	**β3–γ2/γ2**	**Triple/β2–γ2**	**Triple/β3–γ2**	**β2–γ2 or β3–γ2/γ2**
% Denominator	65	45	65	93	69

Top row shows the density of clusters formed in the molecular layer by each marker, whether alone or co-localized with one or two other markers, as indicated (mean±s.d., *n*=5 mice). Bottom row shows the fraction of clusters containing the indicated co-localization of markers relative to the marker in the denominator. Clusters containing the γ2-subunit are considered to be postsynaptic.

## References

[b1] Eccles J. C. Circuits in the cerebellar control of movement. Proc. Natl Acad. Sci. USA 58, 336–343 (1967). 5231614 10.1073/pnas.58.1.336 PMC335638

[b2] Marr D. A theory of cerebellar cortex. J. Physiol. 202, 437–470 (1969). 5784296 10.1113/jphysiol.1969.sp008820 PMC1351491

[b3] Ito M. The modifiable neuronal network of the cerebellum. Jpn J. Physiol. 34, 781–792 (1984). 6099855 10.2170/jjphysiol.34.781

[b4] Llinas R. & Sugimori M. Electrophysiological properties of in vitro Purkinje cell dendrites in mammalian cerebellar slices. J. Physiol. 305, 197–213 (1980). 7441553 10.1113/jphysiol.1980.sp013358 PMC1282967

[b5] Womack M. & Khodakhah K. Active contribution of dendrites to the tonic and trimodal patterns of activity in cerebellar Purkinje neurons. J. Neurosci. 22, 10603–10612 (2002). 12486152 10.1523/JNEUROSCI.22-24-10603.2002 PMC6758439

[b6] Jaeger D. & Bower J. M. Synaptic control of spiking in cerebellar Purkinje cells: dynamic current clamp based on model conductances. J. Neurosci. 19, 6090–6101 (1999). 10407045 10.1523/JNEUROSCI.19-14-06090.1999 PMC6783079

[b7] De Schutter E. & Bower J. M. Simulated responses of cerebellar Purkinje cells are independent of the dendritic location of granule cell synaptic inputs. Proc. Natl Acad. Sci. USA 91, 4736–4740 (1994). 8197127 10.1073/pnas.91.11.4736 PMC43863

[b8] Oldfield C. S., Marty A. & Stell B. M. Interneurons of the cerebellar cortex toggle Purkinje cells between up and down states. Proc. Natl Acad. Sci. USA 107, 13153–13158 (2010). 20615960 10.1073/pnas.1002082107 PMC2919942

[b9] Mittmann W., Koch U. & Hausser M. Feed-forward inhibition shapes the spike output of cerebellar Purkinje cells. J. Physiol. 563, 369–378 (2005). 15613376 10.1113/jphysiol.2004.075028 PMC1665592

[b10] Hausser M. & Clark B. A. Tonic synaptic inhibition modulates neuronal output pattern and spatiotemporal synaptic integration. Neuron 19, 665–678 (1997). 9331356 10.1016/s0896-6273(00)80379-7

[b11] Conti R., Tan Y. P. & Llano I. Action potential-evoked and ryanodine-sensitive spontaneous Ca^2+^ transients at the presynaptic terminal of a developing CNS inhibitory synapse. J. Neurosci. 24, 6946–6957 (2004). 15295030 10.1523/JNEUROSCI.1397-04.2004 PMC6729609

[b12] Llano I. . Presynaptic calcium stores underlie large-amplitude miniature IPSCs and spontaneous calcium transients. Nat. Neurosci. 3, 1256–1265 (2000). 11100146 10.1038/81781

[b13] Vincent P. & Marty A. Fluctuations of inhibitory postsynaptic currents in Purkinje cells from rat cerebellar slices. J. Physiol. 494, 183–199 (1996). 8814615 10.1113/jphysiol.1996.sp021484 PMC1160623

[b14] Kano M., Rexhausen U., Dreessen J. & Konnerth A. Synaptic excitation produces a long-lasting rebound potentiation of inhibitory synaptic signals in cerebellar Purkinje cells. Nature 356, 601–604 (1992). 1313949 10.1038/356601a0

[b15] Brandon N., Jovanovic J. & Moss S. Multiple roles of protein kinases in the modulation of γ-aminobutyric acid_A_ receptor function and cell surface expression. Pharmacol. Ther. 94, 113–122 (2002). 12191597 10.1016/s0163-7258(02)00175-4

[b16] McDonald B. J. & Moss S. J. Differential phosphorylation of intracellular domains of γ- aminobutyric acid type A receptor subunits by calcium/calmodulin type 2- dependent protein kinase and cGMP-dependent protein kinase. J. Biol. Chem. 269, 18111–18117 (1994). 8027073

[b17] McDonald B. J. & Moss S. J. Conserved phosphorylation of the intracellular domains of GABA_A_ receptor β2 and β3 subunits by cAMP-dependent protein kinase, cGMP-dependent protein kinase protein kinase C and Ca^2+^/calmodulin type II-dependent protein kinase. Neuropharmacol 36, 1377–1385 (1997). 10.1016/s0028-3908(97)00111-1 9423925

[b18] Moss S. J. & Smart T. G. Constructing inhibitory synapses. Nat. Rev. Neurosci. 2, 240–250 (2001). 11283747 10.1038/35067500

[b19] Houston C. M., Lee H. H. C., Hosie A. M., Moss S. J. & Smart T. G. Identification of the sites for CaMK-II-dependent phosphorylation of GABA_A_ receptors. J. Biol. Chem. 282, 17855–17865 (2007). 17442679 10.1074/jbc.M611533200

[b20] Kano M., Kano M., Fukunaga K. & Konnerth A. Ca^2+^-induced rebound potentiation of γ-aminobutyric acid-mediated currents requires activation of Ca^2+^/calmodulin-dependent kinase II. Proc. Natl Acad. Sci. USA 93, 13351–13356 (1996). 8917594 10.1073/pnas.93.23.13351 PMC24096

[b21] Kano M. & Konnerth A. Potentiation of GABA-mediated currents by cAMP-dependent protein kinase. Neuroreport 3, 563–566 (1992). 1421107 10.1097/00001756-199207000-00004

[b22] Kawaguchi S. Y. & Hirano T. Signaling cascade regulating long-term potentiation of GABA_A_ receptor responsiveness in cerebellar Purkinje neurons. J. Neurosci. 22, 3969–3976 (2002). 12019316 10.1523/JNEUROSCI.22-10-03969.2002 PMC6757657

[b23] Moss S. J. & Smart T. G. Modulation of amino acid-gated ion channels by protein phosphorylation. Int. Rev. Neurobiol. 39, 1–52 (1996). 8894843 10.1016/s0074-7742(08)60662-5

[b24] Kittler J. T. & Moss S. J. Modulation of GABA_A_ receptor activity by phosphorylation and receptor trafficking: implications for the efficacy of synaptic inhibition. Curr. Opin. Neurobiol. 13, 341–347 (2003). 12850219 10.1016/s0959-4388(03)00064-3

[b25] Fritschy J. M. . Five subtypes of type A γ-aminobutyric acid receptors identified in neurons by double and triple immunofluorescence staining with subunit- specific antibodies. Proc. Natl Acad. Sci. USA 89, 6726–6730 (1992). 1323116 10.1073/pnas.89.15.6726 PMC49576

[b26] Pirker S., Schwarzer C., Wieselthaler A., Sieghart W. & Sperk G. GABA_A_ receptors: immunocytochemical distribution of 13 subunits in the adult rat brain. Neuroscience 101, 815–850 (2000). 11113332 10.1016/s0306-4522(00)00442-5

[b27] Poltl A., Hauer B., Fuchs K., Tretter V. & Sieghart W. Subunit composition and quantitative importance of GABA_A_ receptor subtypes in the cerebellum of mouse and rat. J. Neurochem. 87, 1444–1455 (2003). 14713300 10.1046/j.1471-4159.2003.02135.x

[b28] Miralles C. P., Li M., Mehta A. K., Khan Z. U. & De Blas A. L. Immunocytochemical localization of the β_3_ subunit of the γ-aminobutyric acid_A_ receptor in the rat brain. J. Comp Neurol. 413, 535–548 (1999). 10495441

[b29] Duguid I. C. & Smart T. G. Retrograde activation of presynaptic NMDA receptors enhances GABA release at cerebellar interneuron-Purkinje cell synapses. Nat. Neurosci. 7, 525–533 (2004). 15097992 10.1038/nn1227

[b30] Hashimoto T., Ishii T. & Ohmori H. Release of Ca^2+^ is the crucial step for the potentiation of IPSCs in the cultured cerebellar Purjinke cells of the rat. J. Physiol. 497, 611–627 (1996). 9003548 10.1113/jphysiol.1996.sp021794 PMC1160959

[b31] Laurie D. J., Wisden W. & Seeburg P. H. The distribution of thirteen GABA_A_ receptor subunit mRNAs in the rat brain. III. Embryonic and postnatal development. J. Neurosci. 12, 4151–4172 (1992). 1331359 10.1523/JNEUROSCI.12-11-04151.1992 PMC6576006

[b32] Briatore F., Patrizi A., Viltono L., Sassoe-Pognetto M. & Wulff P. Quantitative organization of GABAergic synapses in the molecular layer of the mouse cerebellar cortex. PLoS ONE 5, e12119 (2010). 20711348 10.1371/journal.pone.0012119 PMC2920831

[b33] Sur C. . Loss of the major GABA_A_ receptor subtype in the brain is not lethal in mice. J. Neurosci. 21, 3409–3418 (2001). 11331371 10.1523/JNEUROSCI.21-10-03409.2001 PMC6762474

[b34] Houston C. M., Hosie A. M. & Smart T. G. Distinct regulation of β2 and β3 subunit-containing cerebellar synaptic GABA_A_ receptors by calcium/calmodulin-dependent protein kinase II. J. Neurosci. 28, 7574–7584 (2008). 18650335 10.1523/JNEUROSCI.5531-07.2008 PMC6670840

[b35] Thompson S. A. . Salicylidene salicylhydrazide, a selective inhibitor of β1-containing GABA_A_ receptors. Br. J. Pharmacol. 142, 97–106 (2004). 15100159 10.1038/sj.bjp.0705689 PMC1574914

[b36] Belelli D., Lambert J. J., Peters J. A., Wafford K. & Whiting P. J. The interaction of the general anesthetic etomidate with the γ-aminobutyric acid type A receptor is influenced by a single amino acid. Proc. Natl Acad. Sci. USA 94, 11031–11036 (1997). 9380754 10.1073/pnas.94.20.11031 PMC23576

[b37] Chang B. H., Mukherji S. & Soderling T. R. Characterization of a calmodulin kinase II inhibitor protein in brain. Proc. Natl Acad. Sci. USA 95, 10890–10895 (1998). 9724800 10.1073/pnas.95.18.10890 PMC27991

[b38] McDonald B. J. . Adjacent phosphorylation sites on GABA_A_ receptor β subunits determine regulation by cAMP-dependent protein kinase. Nat. Neurosci. 1, 23–28 (1998). 10195104 10.1038/223

[b39] Houston C. M., He Q. & Smart T. G. CaMKII phosphorylation of the GABA_A_ receptor: receptor subtype- and synapse-specific modulation. J. Physiol. 587, 2115–2125 (2009). 19332484 10.1113/jphysiol.2009.171603 PMC2697286

[b40] Traynelis S. F., Silver R. A. & Cull-Candy S. G. Estimated conductance of glutamate receptor channels activated during EPSCs at the cerebellar mossy fiber-granule cell synapse. Neuron 11, 279–289 (1993). 7688973 10.1016/0896-6273(93)90184-s

[b41] Lledo P. M., Zhang X., Sudhof T. C., Malenka R. C. & Nicoll R. A. Postsynaptic membrane fusion and long-term potentiation. Science 279, 399–403 (1998). 9430593 10.1126/science.279.5349.399

[b42] Sudhof T. C. The synaptic vesicle cycle: a cascade of protein-protein interactions. Nature 375, 645–653 (1995). 7791897 10.1038/375645a0

[b43] Schiavo G., Matteoli M. & Montecucco C. Neurotoxins affecting neuroexocytosis. Physiol. Rev. 80, 717–766 (2000). 10747206 10.1152/physrev.2000.80.2.717

[b44] Mollenhauer H. H., Morre D. J. & Rowe L. D. Alteration of intracellular traffic by monensin; mechanism, specificity and relationship to toxicity. Biochim. Biophys. Acta 1031, 225–246 (1990). 2160275 10.1016/0304-4157(90)90008-Z PMC7148783

[b45] Charych E. I. . The brefeldin A-inhibited GDP/GTP exchange factor 2, a protein involved in vesicular trafficking, interacts with the beta subunits of the GABA receptors. J. Neurochem. 90, 173–189 (2004). 15198677 10.1111/j.1471-4159.2004.02481.x

[b46] Frey U. & Morris R. G. Synaptic tagging and long-term potentiation. Nature 385, 533–536 (1997). 9020359 10.1038/385533a0

[b47] Bao J., Reim K. & Sakaba T. Target-dependent feedforward inhibition mediated by short-term synaptic plasticity in the cerebellum. J. Neurosci. 30, 8171–8179 (2010). 20554867 10.1523/JNEUROSCI.0276-10.2010 PMC6634587

[b48] Houston C. M. & Smart T. G. CaMK-II modulation of GABA_A_ receptors expressed in HEK293, NG108-15 and rat cerebellar granule neurons. Eur. J. Neurosci. 24, 2504–2514 (2006). 17100839 10.1111/j.1460-9568.2006.05145.x

[b49] Galante M. & Marty A. Presynaptic ryanodine-sensitive calcium stores contribute to evoked neurotransmitter release at the basket cell-Purkinje cell synapse. J. Neurosci. 23, 11229–11234 (2003). 14657182 10.1523/JNEUROSCI.23-35-11229.2003 PMC6741031

[b50] De Koninck Y. & Mody I. Noise analysis of miniature IPSCs in adult rat brain slices: properties and modulation of synaptic GABA_A_ receptor channels. J. Neurophysiol. 71, 1318–1335 (1994). 8035217 10.1152/jn.1994.71.4.1318

[b51] Otis T. S., De Koninck Y. & Mody I. Lasting potentiation of inhibition is associated with an increased number of γ-aminobutyric acid type A receptors activated during miniature inhibitory postsynaptic currents. Proc. Natl Acad. Sci. USA 91, 7698–7702 (1994). 8052645 10.1073/pnas.91.16.7698 PMC44469

[b52] Llano I., Leresche N. & Marty A. Calcium entry increases the sensitivity of cerebellar Purkinje cells to applied GABA and decreases inhibitory synaptic currents. Neuron 6, 565–574 (1991). 2015092 10.1016/0896-6273(91)90059-9

[b53] Eghbali M., Curmi J. P., Birnir B. & Gage P. W. Hippocampal GABA_A_ channel conductance increased by diazepam. Nature 388, 71–75 (1997). 9214504 10.1038/40404

[b54] Wang Q. . Control of synaptic strength, a novel function of Akt. Neuron 38, 915–928 (2003). 12818177 10.1016/s0896-6273(03)00356-8

[b55] Saiepour L. . Complex role of collybistin and gephyrin in GABA_A_ receptor clustering. J. Biol. Chem. 285, 29623–29631 (2010). 20622020 10.1074/jbc.M110.121368 PMC2937993

[b56] Thomas P., Mortensen M., Hosie A. M. & Smart T. G. Dynamic mobility of functional GABA_A_ receptors at inhibitory synapses. Nat. Neurosci. 8, 889–897 (2005). 15951809 10.1038/nn1483

[b57] Bannai H. . Activity-dependent tuning of inhibitory neurotransmission based on GABA_A_R diffusion dynamics. Neuron 62, 670–682 (2009). 19524526 10.1016/j.neuron.2009.04.023

[b58] Vetiska S. M. . GABA_A_ receptor-associated phosphoinositide 3-kinase is required for insulin-induced recruitment of postsynaptic GABA_A_ receptors. Neuropharmacol 52, 146–155 (2007). 10.1016/j.neuropharm.2006.06.023 16890252

[b59] Wan Q. . Recruitment of functional GABA_A_ receptors to postsynaptic domains by insulin. Nature 388, 686–690 (1997). 9262404 10.1038/41792

[b60] Kawaguchi S. Y. & Hirano T. Sustained structural change of GABA_A_ receptor-associated protein underlies long-term potentiation at inhibitory synapses on a cerebellar Purkinje neuron. J. Neurosci. 27, 6788–6799 (2007). 17581966 10.1523/JNEUROSCI.1981-07.2007 PMC6672699

[b61] Mann-Metzer P. & Yarom Y. Electrotonic coupling interacts with intrinsic properties to generate synchronized activity in cerebellar networks of inhibitory interneurons. J. Neurosci. 19, 3298–3306 (1999). 10212289 10.1523/JNEUROSCI.19-09-03298.1999 PMC6782243

[b62] Callaway J. C., Lasser-Ross N. & Ross W. N. IPSPs strongly inhibit climbing fiber-activated [Ca^2+^]i increases in the dendrites of cerebellar Purkinje neurons. J. Neurosci. 15, 2777–2787 (1995). 7722628 10.1523/JNEUROSCI.15-04-02777.1995 PMC6577758

[b63] Brunel N., Hakim V., Isope P., Nadal J. P. & Barbour B. Optimal information storage and the distribution of synaptic weights: perceptron versus Purkinje cell. Neuron 43, 745–757 (2004). 15339654 10.1016/j.neuron.2004.08.023

[b64] Welsh J. P., Lang E. J., Suglhara I. & Llinas R. Dynamic organization of motor control within the olivocerebellar system. Nature 374, 453–457 (1995). 7700354 10.1038/374453a0

[b65] Ozden I., Sullivan M. R., Lee H. M. & Wang S. S. Reliable coding emerges from coactivation of climbing fibers in microbands of cerebellar Purkinje neurons. J. Neurosci. 29, 10463–10473 (2009). 19710300 10.1523/JNEUROSCI.0967-09.2009 PMC2783593

[b66] Schultz S. R., Kitamura K., Post-Uiterweer A., Krupic J. & Hausser M. Spatial pattern coding of sensory information by climbing fiber-evoked calcium signals in networks of neighboring cerebellar Purkinje cells. J. Neurosci. 29, 8005–8015 (2009). 19553440 10.1523/JNEUROSCI.4919-08.2009 PMC6666035

[b67] Apps R. & Garwicz M. Anatomical and physiological foundations of cerebellar information processing. Nat. Rev. Neurosci. 6, 297–311 (2005). 15803161 10.1038/nrn1646

[b68] McKay B. E. & Turner R. W. Physiological and morphological development of the rat cerebellar Purkinje cell. J. Physiol. 567, 829–850 (2005). 16002452 10.1113/jphysiol.2005.089383 PMC1474219

[b69] Wierenga C. J. . Molecular and electrophysiological characterization of GFP-expressing CA1 interneurons in GAD65-GFP mice. PLoS ONE 5, e15915 (2010). 21209836 10.1371/journal.pone.0015915 PMC3013138

[b70] Notter T., Panzanelli P., Pfister S., Mircsof D. & Fritschy J. M. A protocol for concurrent high-quality immunohistochemical and biochemical analyses in adult mouse central nervous system. Eur. J. Neurosci. 39, 165–175 (2014). 24325300 10.1111/ejn.12447

