# Supplementary material for: Interneuron- and GABAA receptor-specific inhibitory synaptic plasticity in cerebellar Purkinje cells
Source: Nat Commun. 2015 Jul 16;6:7364. doi: 10.1038/ncomms8364 (PMC4518301; doi:10.1038/ncomms8364)
Supplement: Supplementary Information — Supplementary Figures 1-12 and Supplementary Tables 1-3 [file ncomms8364-s1.pdf]

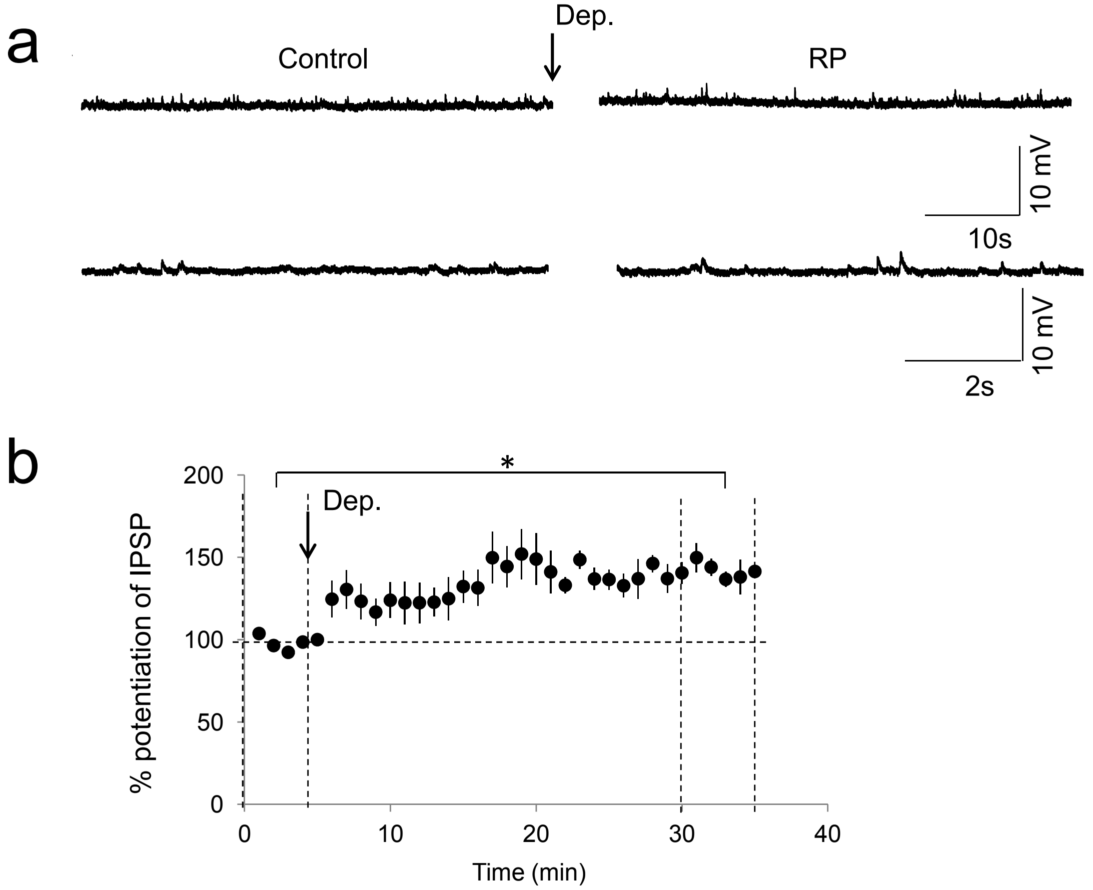

Supplementary Figure 1. Rebound potentiation of IPSPs in PCs.

a, IPSPs recorded with a K^+ gluconate pipette solution in current clamp mode also exhibited similar potentiation after depolarization of Purkinje cells. A holding current was injected to keep the membrane potential hyperpolarized at -70mV to avoid spontaneous spiking. Arrow indicates the time point of depolarization.

b, Time profiles for IPSP amplitudes in control and after RP induction by depolarisation (Dep; arrow). All events are normalised to mean values calculated from IPSPs recorded over 1 min before applying the stimulus (t = 0). All points are mean ± sem (n = 7). **P*<0.05, paired *t*-test comparing IPSPs in the periods indicated by the vertical dotted lines.

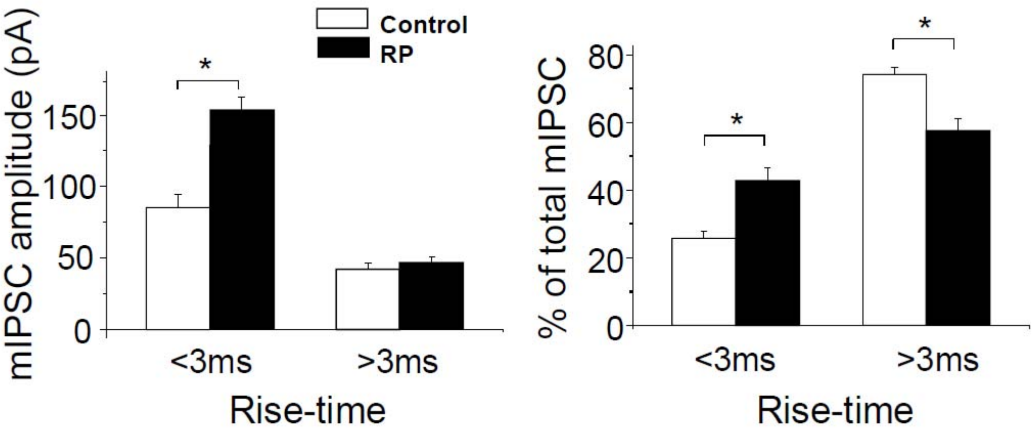

Supplementary Figure 2. Properties of IPSCs during RP.

Bargraphs of mIPSC amplitudes (left panel) grouped according to rise times (<3 ms or >3 ms) in control and after the induction of RP. Relative proportion of fast (<3 ms) and slow rise-time (>3 ms) grouped mIPSCs before and after RP (right panel). Bars are means ± sem from n = 7; *$P<0.05$, paired *t*-test.

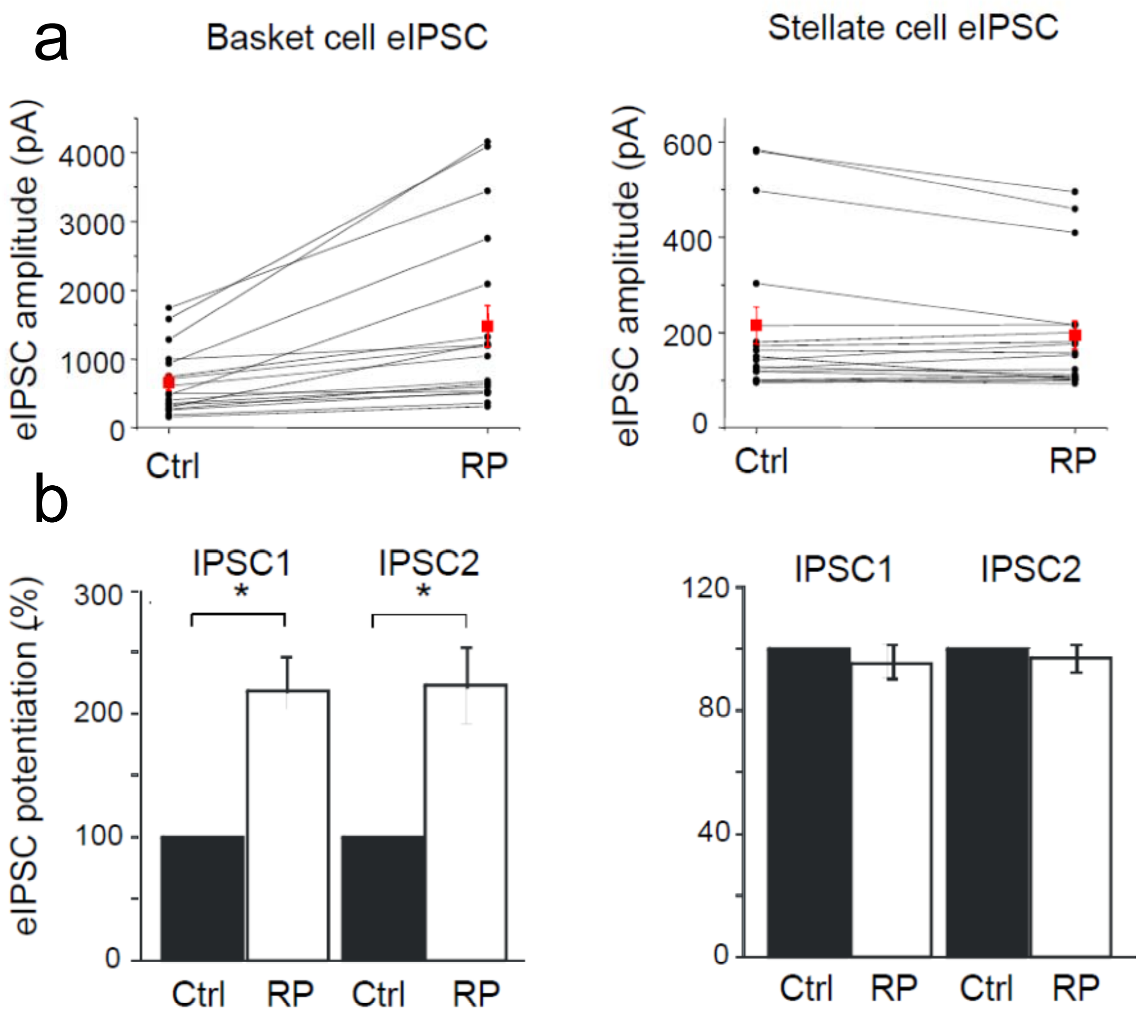

Supplementary Figure 3. IN-PC paired recording.

a, Paired stimulation of IN soma evokes IPSCs in PCs under control conditions and following the induction of RP (individual IPSC pairs - black circles; mean IPSC pairs – red squares) for basket cell - PC (left) and stellate cell - PC (right) synapses.

b, Bargraphs of relative potentiation of paired eIPSCs from BC (left) and SC (right) inputs following RP induction (n = 10, *P < 0.05, paired *t*-test).

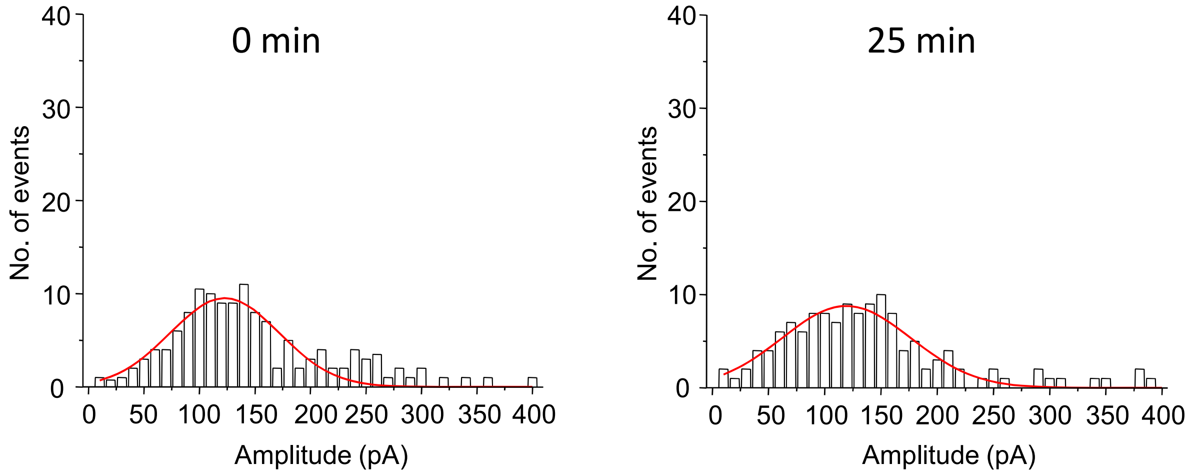

Supplementary Figure 4. Stability of mIPSCs at SC-PC synapses.

GABA synaptic current amplitude distribution generated from a single PC recording mIPSCs at SC-PC synapses (identified by rise-times >3 ms). The distributions are fit by a single Gaussian function (red line). Note the mIPSC amplitude stability over a 25 min recording period.

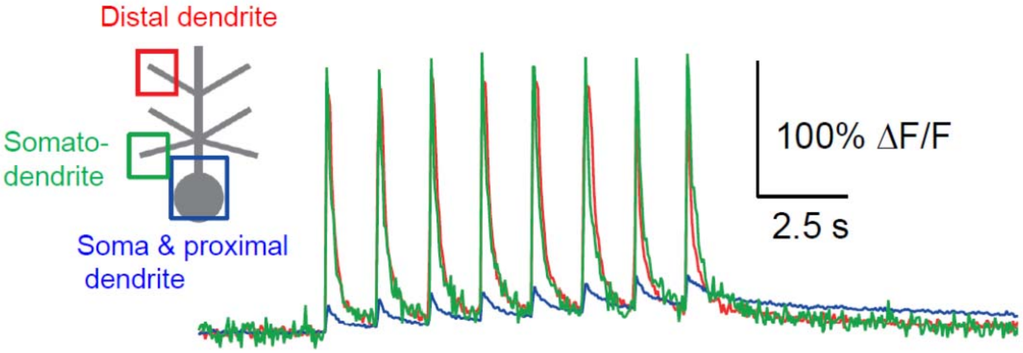

Supplementary Figure 5. Ca^{2+} transients during RP induction are similar in proximal and distal spiny branchlets.

Line scans of Ca^{2+} transients occurring at two dendritic spiny branchlets on the same cell at different locations in the molecular layer (shown by schematic). Similar Ca^{2+} entry was seen in distal- (red) and somato-dendritic (green) branch locations. Somatic Ca^{2+} transients are shown in blue.

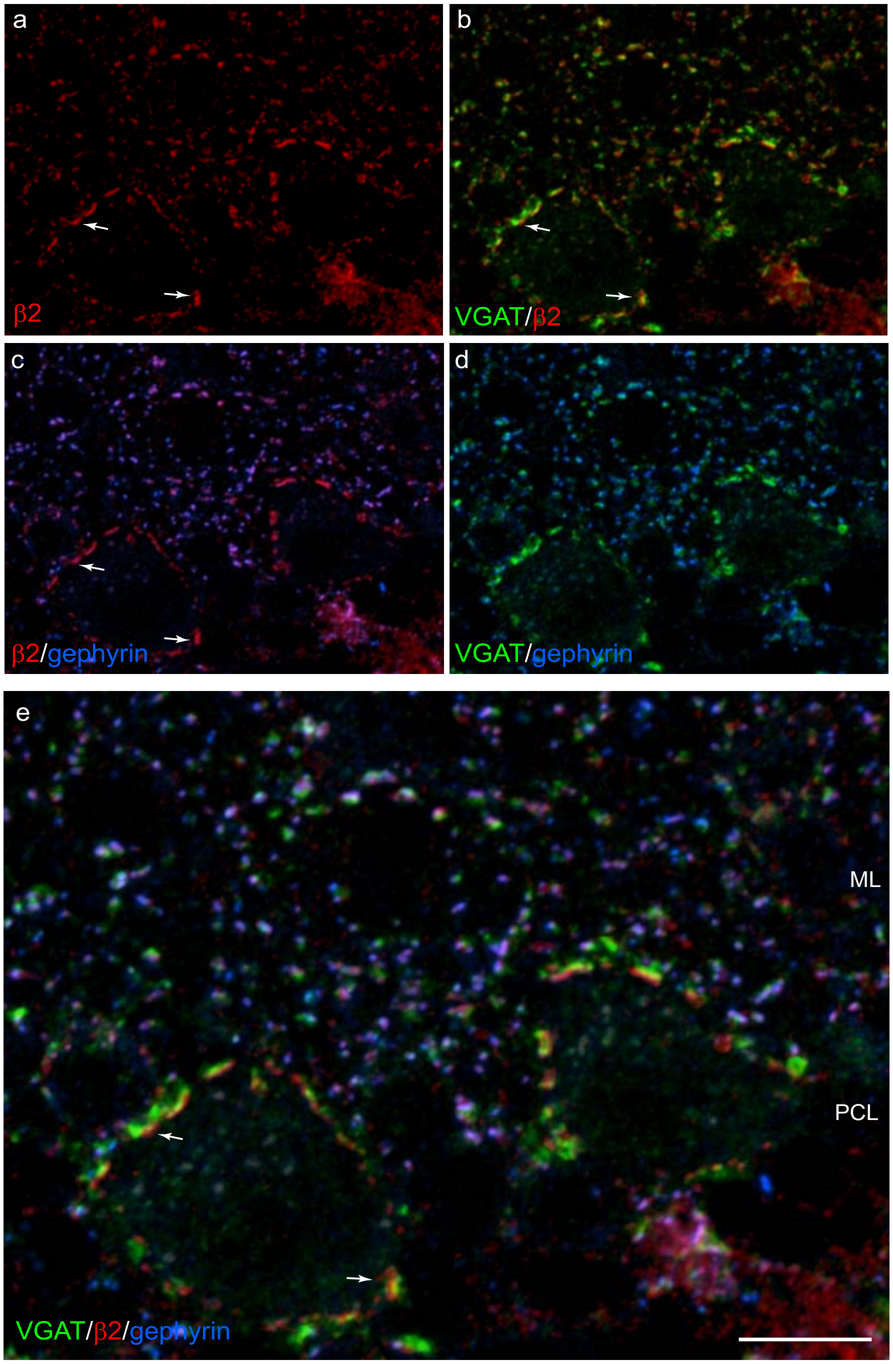

Supplementary Figure 6. Postsynaptic localization of β2 subunit staining in the cerebellum. This is illustrated by triple labelling in conjunction with the vesicular GABA transporter (VGAT) and gephyrin. **a**, Clustered distribution of β2 subunits (red) around Purkinje cell (PCL – see panel **e**) somata (arrows) and molecular layers (ML). **b**, Double labelling for VGAT (green) reveals the apposition of most β2 subunit clusters to a GABAergic presynaptic terminal. **c**, Double labelling for gephyrin shows widespread colocalization of both markers within individual clusters (magenta); note the characteristic absence of gephyrin staining around PC somata, where β2 subunit clusters appear single-labelled (arrows). **d,** Double labelling for VGAT and gephyrin confirms the apposition between these pre- and postsynaptic markers. **e**, Combining the three channels together confirms that the vast majority of β2 subunit clusters are postsynaptic.

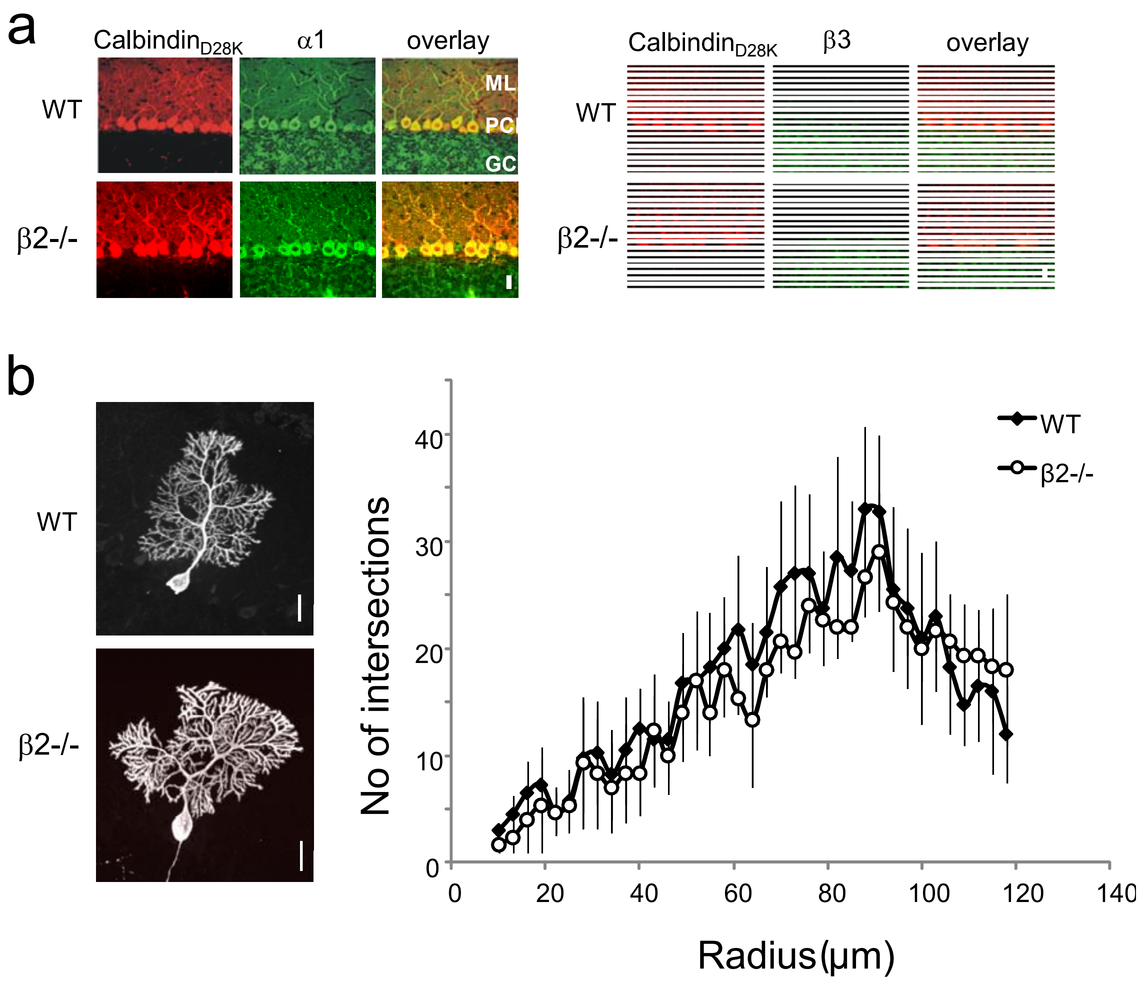

Supplementary Figure 7. GABA$_A$R subunit expression and PC morphology in β2-/- and WT cerebellar slices. a, Panels show the expression levels of α and β subunits in WT and β2-/- PCs. CalbindinD28K immunostaining (left columns, red) was used as a PC marker; green (middle columns) indicates antibody staining against individual α and β subunits; and overlays are shown in the right columns. GC - granule cell layer, PC – Purkinje cell layer, ML – Molecular layer. **b,** Lower panels: Left, individual PCs from WT and β2 -/- animals reconstructed after filling with biocytin via the patch pipette. Scale bar is 20 μm. Right, Sholl analysis performed for WT (n = 4) and β2-/- PCs (n = 3).

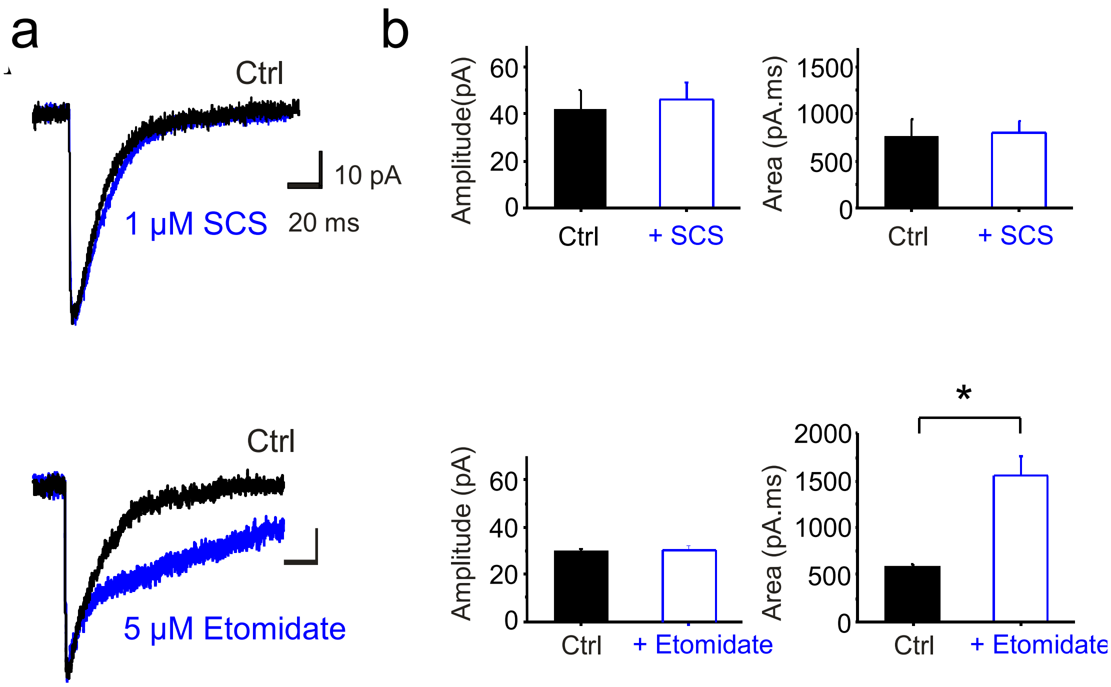

Supplementary Figure 8. Pharmacology of PC GABA$_A$ receptors in β2-/- slices.

a, Left, superimposed peak-scaled average mIPSCs in the presence (blue) and absence (black) of either 1 μM SCS (upper) or 5 μM etomidate (lower) in β2-/- PCs.

b, Bargraphs of mean ± sem for mIPSC amplitudes and areas in control (Ctrl), and in the presence of SCS (upper) or etomidate (lower). n = 5 for both, *$P < 0.05$, paired *t*-test.

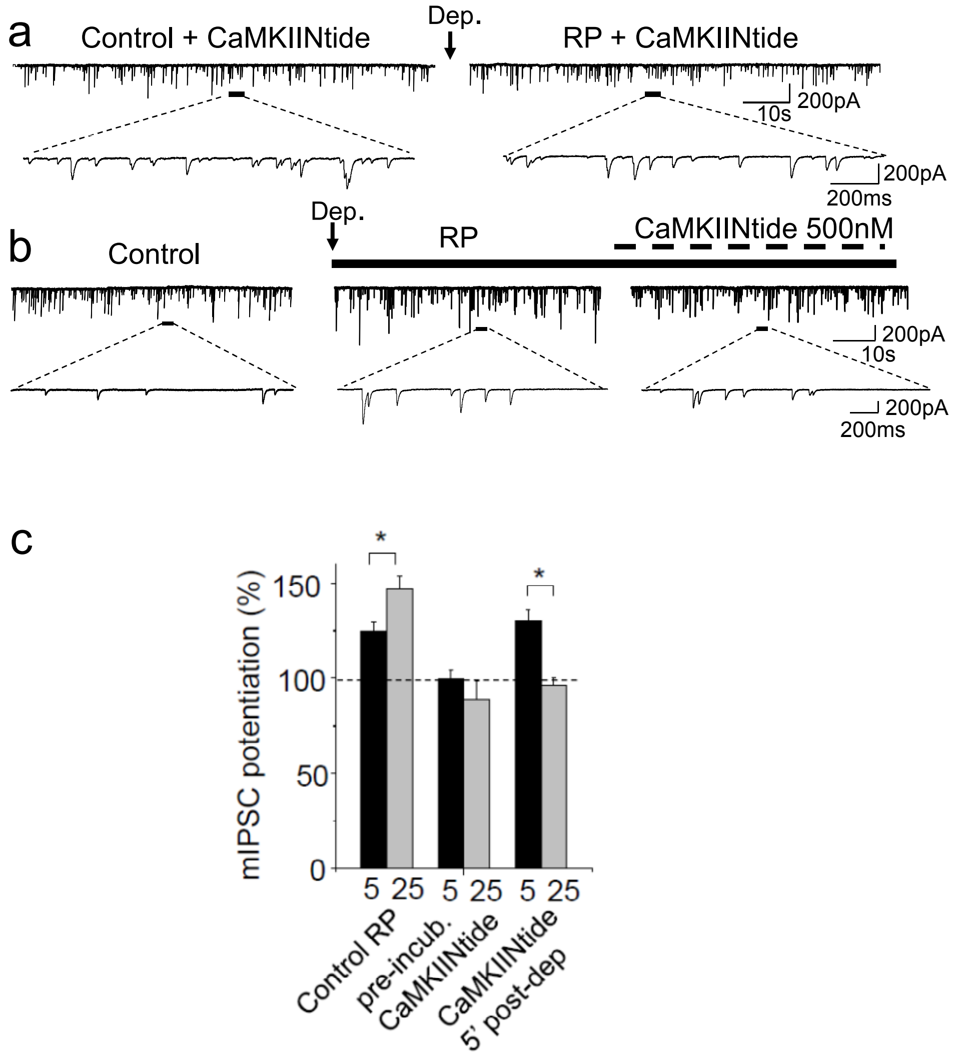

Supplementary Figure 9. CaMKIINtide inhibits the induction and maintenance of RP.

a, Recordings of mIPSCs before (left, t = 0') and after RP induction (right, t = 25') in the presence of 500 nM CaMKIINtide. High resolution mIPSC recordings are shown below and taken from the corresponding time points indicated.

b, mIPSCs recorded under control conditions, after RP induction and during the application of 500 nM CaMKIINtide (dotted line), 5 min after the induction of RP. mIPSCs at high time resolution are shown below for the indicated periods.

c, Bargraph of mIPSC potentiation measured at 5 and 25 min after the induction of RP for: Control RP; pre-incubated in CaMKIINtide; and CaMKIINtide applied 5 min post-stimulus. *P < 0.05, paired *t*-test.

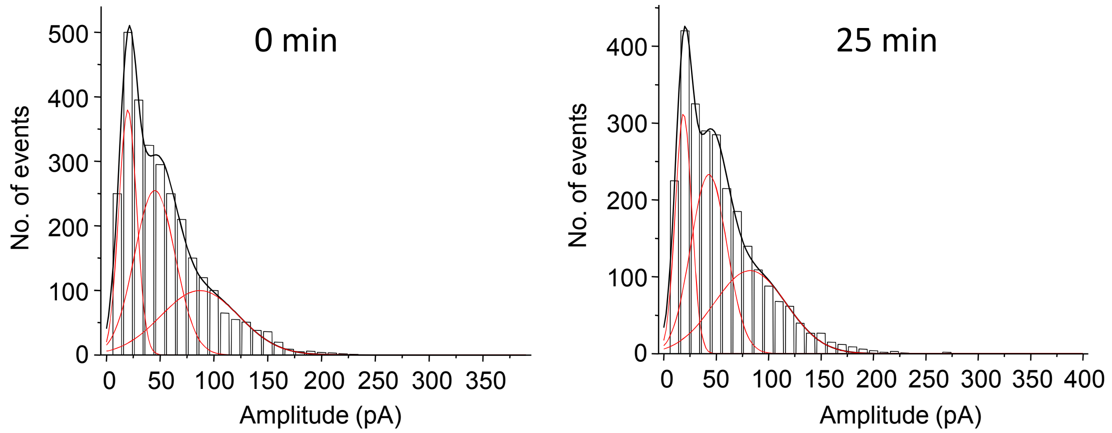

Supplementary Figure 10. Inhibition of CaMKII does not affect basal IPSCs in PCs.
Basal IPSC amplitude distributions assembled for all IPSCs recorded from a typical PC at the start (0 min) and after 25 min in the presence of CaMKIINtide (500 nM), without any depolarization applied to the PC. Individual distributions were fit with Gaussian functions (red lines) and the overall fit is shown by the black line.

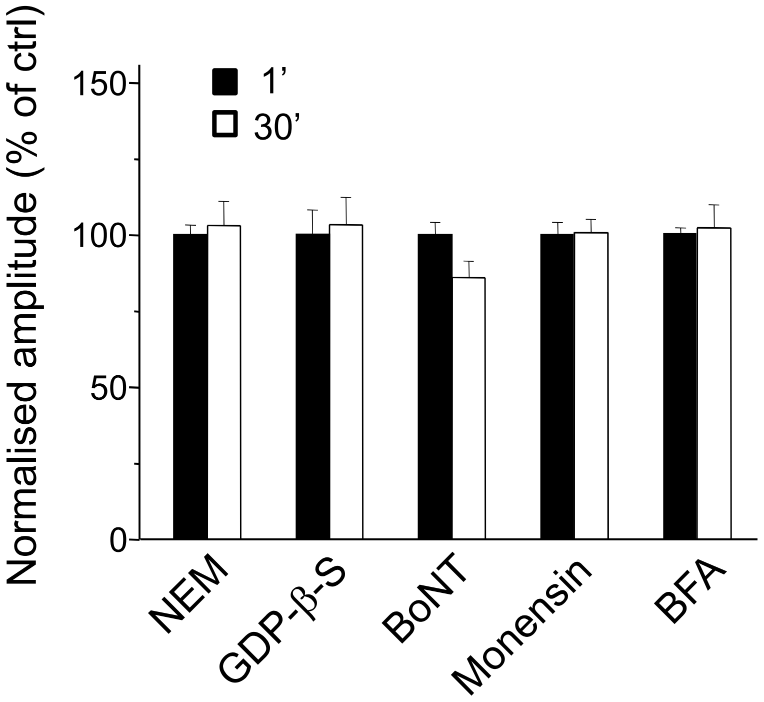

Supplementary Figure 11. Protein trafficking inhibitors do not affect baseline mIPSC amplitude.

Bargraph representing mean ± sem for mIPSC amplitudes measured in PCs at the beginning (1', black bars) and after a 30 min recording period (open bars) in the presence of: 250 μM NEM (n = 4); 1 mM GDP-β-S (n = 5); 500 nM BoNT (n = 5); 75 μM Monensin (n = 5); or 200 nM BFA (n = 6), internally-applied via the patch pipette solution.

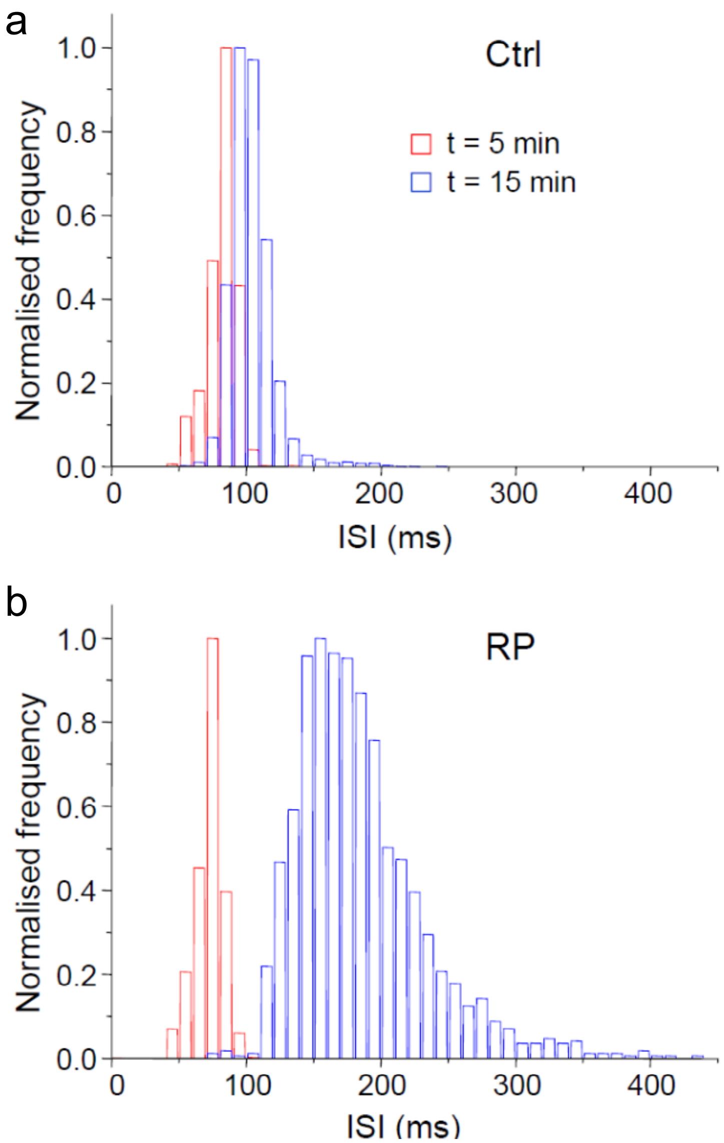

Supplementary Figure 12. Changes to inter-spike intervals after RP induction.
ISI distributions recorded at 5 min (red) and 15 min (blue) after patch breakthrough for PC spike firing under control conditions (**a**) and following the induction of RP (**b**).

Supplementary Table 1 - Properties of IPSCs before and after rebound potentiation

**IPSC property**	**Control**		**RP**	
	**0’**	**30’**	**Pre-**	**Post-25’**
Amplitude (pA)	88.2 ± 15.9	84.9 ± 10.5	75.5± 6.2	120.9 ± 11.9*
Frequency (Hz)	4.9 ± 0.3	5.0 ± 0.5	5.7 ± 1.5	5.3 ± 1.3
Rise time (ms)	2.0 ± 0.2	2.8 ± 0.4	1.2 ± 0.2	1.5 ± 0.1
Decay time (τ_W; ms)	10.3 ± 0.8	16.1 ± 1.8*	10.9 ± 0.5	12.3 ± 0.8

Supplementary Table 1. Properties of IPSCs recorded from PCs before (control) and after the induction of RP. * indicates $P < 0.05$ for RP values from the equivalent timed measurements in control.

Supplementary Table 2 - Quantification of Dystrophin, β2 and γ2 subunit clusters

Marker	γ2	β2	Dys	Dys-β2	β2-γ2	Dys-γ2	Triple	β2/γ2 no Dys	Dys/γ2 no β2
Mean / 1000 μm^2	142 ± 13	133 ± 12	69 ± 7	54 ± 8	98 ± 11	54 ± 6	51 ± 7	47	3

Marker	β2-γ2/γ2	Dys-γ2/γ2	Triple/β2-γ2	Triple/dys-γ2	β2-γ2 or Dys-γ2/γ2
% denominator	69	42	56	93	71

Supplementary Table 3 - Quantification of β2, β3 and γ2 subunit clusters

Marker	γ2	β2	β3	β2/β3	β2/γ2	β3/γ2	Triple	β2γ2 no β3	β3γ2 no β2
Mean/ 1000 μm^2	137 ± 28	123 ± 15	96 ± 26	64 ± 14	90 ± 16	61 ± 16	58 ± 14	33	3

Marker	β2-γ2/γ2	β3-γ2/γ2	Triple/β2-γ2	Triple/β3-γ2	β2-γ2 or β3-γ2/γ2
% denominator	65	45	65	93	69

Supplementary Tables 2 & 3. Top rows show the density of clusters formed in the molecular layer by each marker, whether alone or co-localised with one or two other markers, as indicated (mean ± SD, n = 5 mice). Bottom rows show the fraction of clusters containing the indicated co-localization of markers relative to the marker in the denominator. Clusters containing the γ2 subunit are considered to be postsynaptic.
